# Molecular rationale for SARS-CoV-2 spike circulating mutations able to escape bamlanivimab and etesevimab monoclonal antibodies

**DOI:** 10.1038/s41598-021-99827-3

**Published:** 2021-10-12

**Authors:** Erik Laurini, Domenico Marson, Suzana Aulic, Alice Fermeglia, Sabrina Pricl

**Affiliations:** 1 Molecular Biology and Nanotechnology Laboratory (MolBNL@UniTS), DEA, University of Trieste, 34127 Trieste, Italy; 2 Department of General Biophysics, Faculty of Biology and Environmental Protection, University of Lodz, 90-136 Lodz, Poland

**Keywords:** Computational biology and bioinformatics, Molecular dynamics

## Abstract

The purpose of this work is to provide an *in silico* molecular rationale of the role eventually played by currently circulating mutations in the receptor binding domain of the SARS-CoV-2 spike protein (S-RBD_CoV‑2_) in evading the immune surveillance effects elicited by the two Eli Lilly LY-CoV555/bamlanivimab and LY-CoV016/etesevimab monoclonal antibodies. The main findings from this study show that, compared to the wild-type SARS-CoV-2 spike protein, mutations E484A/G/K/Q/R/V, Q493K/L/R, S494A/P/R, L452R and F490S are predicted to be markedly resistant to neutralization by LY-CoV555, while mutations K417E/N/T, D420A/G/N, N460I/K/S/T, T415P, and Y489C/S are predicted to confer LY-CoV016 escaping advantage to the viral protein. A challenge of our global *in silico* results against relevant experimental data resulted in an overall 90% agreement. Thus, the results presented provide a molecular-based rationale for all relative experimental findings, constitute a fast and reliable tool for identifying and prioritizing all present and newly reported circulating spike SARS-CoV-2 variants with respect to antibody neutralization, and yield substantial structural information for the development of next-generation vaccines and monoclonal antibodies more resilient to viral evolution.

## Introduction

The 2019 Coronavirus disease (COVID-19)^1,2^ elicited by the novel severe acute respiratory syndrome coronavirus 2 (SARS-CoV-2)^3^ has produced 220,904,838 of confirmed infections globally, and caused 4,570,946 deaths by September 7, 2021^4^. This pandemic has also forced much of the world to enter an unprecedented sort of stand-by condition, with exceptional life-threating situations and unparalleled damage to the global economy. The ability of science and technology to deliver an effective, global solution to COVID-19 will be critical to restoring some semblance of normalcy, and the scientific community has responded commendably to this vital call. In particular, incomparable efforts have been and still are currently focused on the development of effective measures to further limit the spreading of SARS-CoV-2 infection and to treat already affected individuals. To date, drug development is under way; however, no proven effective therapies for this virus currently exist^5^, while drugs that target the dysregulated cytokine responses (aka cytokine storms) characteristic of COVID-19 are available^6^, although their clinical benefit is still a matter of debate^7^. Meanwhile, different mRNA- or virus-based vaccines have received approval (and more are under clinical trial) and have so far provided effective an efficient protection against the disease^8,9^, making vaccination the key weapon in fighting the COVID-19 pandemic. Another promising approach is the isolation of SARS-CoV-2 neutralizing monoclonal antibodies (mAbs)^10,11^. mAbs are immuno-therapeutics, which could (i) potentially deliver immediate benefit in COVID-19 treatment, (ii) act as passive prophylaxis until vaccines become globally available, and (iii) serve as alternative therapeutic strategies in those populations where vaccines have been found to be less protective^11,12^. The recent findings that ansuvimab (mAb114) is a safe and effective treatment for symptomatic infection with Ebola virus is a notable example of the successful use of mAb therapy during an outbreak of infectious disease^13^.

Ab-based therapeutics directed against SARS-CoV-2 still present lights and shadows^14^. Preclinical data and phase-III clinical studies indicate that mAbs could be effectively deployed for prevention or treatment during the viral symptoms phase of the disease^15^. Cocktail formulations of two or more mAbs are preferred over single Ab preparations because these combinations may result in increased antiviral efficacy and viral escape prevention^16–18^. However, Ab cocktails are complicate formulations^19,20^, and such approach likely involves increased production costs and quantities at a time when the supply chain is being pressured into meeting the high demand for COVID-19 therapeutics, vaccines, and therapeutic agents in general.

**Figure 1:**
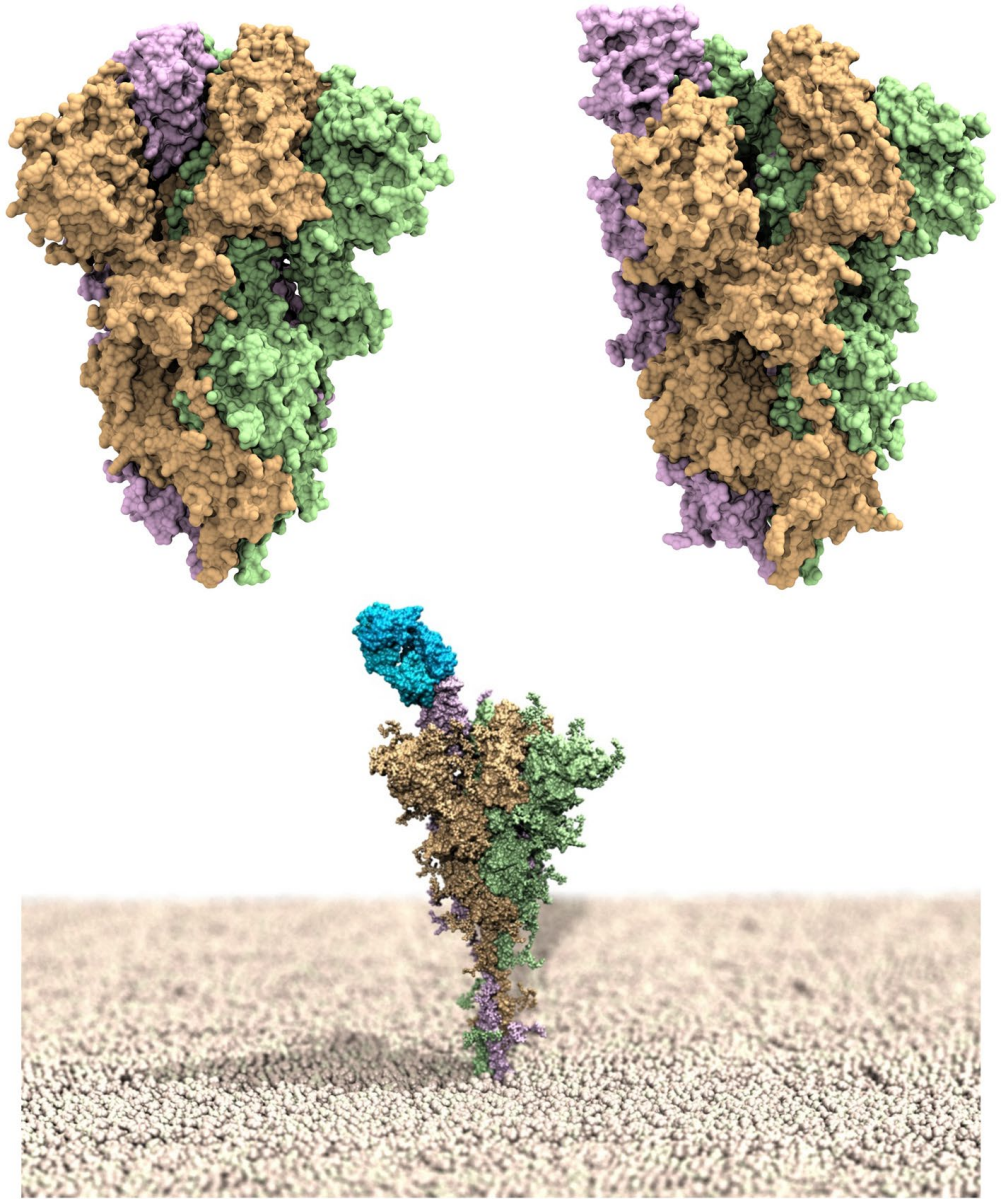
Upper panel: models of the SARS-CoV-2 spike homotrimeric protein in the down (left) and up (right) conformations. The three spike protomers are highlighted by their light green, tan and light purple van der Waals surfaces, respectively. Bottom panel: computer rendering of the full-length SARS-CoV-2 homotrimer embedded in a membrane model (polar heads in light tan spheres), showing one protomer in the up position and in complex with the LY-CoV555 (bamlanivimab) monoclonal antibody (light blue van der Waals surface).

The multi-domain SARS-CoV-2 surface spike (S) protein^21–23^—a trimeric class I fusion protein that mediates viral entry—is the focus of the current Ab discovery efforts. The S protein is composed of two subunits: S1, containing a receptor-binding domain (S-RBD_CoV‑2_) that recognizes and binds the human receptor, the angiotensin-converting enzyme 2 (ACE2)^24–27^, and S2, which mediates viral cell membrane fusion by forming a six-helical bundle via the two-heptad repeat domain. Viral entry is initiated by the upward shift of the spike RBD at the protein's apex which, in turn, promotes ACE2 binding (Fig. 1, top panels). In addition, viral cell entry involves the S-protein priming operated by the cellular transmembrane serine protease 2 (TMPRSS2)^28^, along with other proteases^29^, the removal of subunit S1, and the conformational reorganization of subunit S2; all these processes contribute to viral fusion with the cell and transfers of genetic material following receptor involvement.

Due to the critical nature of the viral S-RBD_CoV‑2_ interaction with ACE2, Abs that bind this domain and interfere with ACE2 attachment can have potent neutralizing activity^30–37^. An S-RBD_CoV‑2_ specific mAb (LY-CoV555 or bamlanivimab) was discovered that can bind the spike RBD in both up (active) (bottom panel in Fig. 1) and down (resting) conformations, and was reported to display high *in vitro* and *in vivo* protection potency, thereby supporting its development as a therapeutic for the treatment and prevention of COVID-19^38^. In the same context, another mAb isolated from a COVID-19 convalescent patient—LY-CoV016 or etesevimab—was soon after reported^39^. This mAb also showed specific SARS-CoV-2 neutralization activity by recognizing another epitope on the S-RBD_CoV‑2_, and was found effective *in vivo* in both prophylactic and therapeutic settings^39^. These two mAbs from Eli Lilly are presently under clinical evaluation for the treatment and prevention of COVID-19, both alone and in cocktail formulations^40–42^. Contextually, in the United States the Food and Drug Administration (FDA) has already granted Emergency Use Authorization (EUA) of the combined bamlanivimab/etesevimab cocktail as anti-SARS-CoV-2 monoclonal antibody therapeutic for the treatment of COVID-19, while the European Medicine Agency (EMA) also recently concluded that these two mAbs can be used together to treat confirmed COVID-19 in patients who do not require supplemental oxygen and who are at high risk of their COVID-19 disease becoming severe. EMA also analyzed the use of LY-CoV555 alone and concluded that, despite uncertainties around the benefits of monotherapy, it could be considered a treatment option.

However, viruses that encode their genome in RNA [e.g*.*, SARS-CoV-2, the Human Immunodeficiency Virus (HIV) and the Influenza A Virus (IAV)], are prone to acquire mutations in time, mainly because of three factors. The first, and likely the most probable source of mutations consists in copying errors as viruses replicate inside host cells^43^. Interestingly, however, this mechanism may be less relevant for SARS-CoV-2 with respect to other RNA viruses, since coronavirus polymerases—*i.e.*, those enzymes the play vital role in viral genome replication and transcription—are endowed with a proofreading mechanism that corrects potentially fatal mistakes^44^. Viral genomic variability may also originate from the recombination of two viral lineages coinfecting the same host^45^. As a third factor, mutations can be induced by the host cell RNA-editing systems, which form part of host natural immunity^46,47^. A further element of complexity is reported in the recent work by Di Giorgio et al.^47^, according to which both the adenosine deaminases acting on RNA (ADAR) and the apolipoprotein B mRNA editing catalytic polypeptide-like (APOBEC) families of proteins are involved in coronavirus genome editing—a process that may change the fate of both virus and patient. Whatever the case, the lesson learned from RNA virus genetics and epidemiology is that mutations are an inevitable consequence of being a virus^48^. Yet, we also know that those mutations that adversely impact any of the vital steps of virus function are swiftly eliminated by natural selection. On the contrary, neutral variations and especially those mutations that endow the virus with a competitive advantage can reach high frequencies.

Thus far, a steadily increasing number of SARS-CoV-2 genetic variants have been emerging and circulating all over the world since the beginning of the COVID-19 pandemic. Although all proteins encoded in the SARS-CoV-2 RNA are continuously reported and catalogued in the plethora of databased and networks dedicated to COVID-19 genomic surveillance^49^, the spike protein is the one more often and constantly reported mutated in the viral genomes sequenced worldwide^50^. In this respect, both the United States Center for Disease Control and Prevention (CDC) and the equivalent European agency (ECDC), in collaboration with the World Health Organization (WHO) and several governmental authorities and working groups have developed a SARS-CoV-2 variant classification schema that groups all major viral variants into main groups: variants of high consequence (VOHC, CDC), variants of concern (VOC), variants of interest (VOI) and variants under monitoring (VUM, ECDC), depending on their associated degree of impact on transmissibility, severity and/or reduced neutralization by antibody/efficacy of Ab treatments (of note, European and US classification may not fully coincide since the importance of variants may differ by location). At present, no SARS-CoV-2 circulating variants have been classified as VOHC, while a substantial number of them have been marked as VOC and VOI/VUM by CDC^51^ and ECDC^52^, and all of them are characterized by the presence of at least one spike mutation of interest (MOI)^52^ (see the “Discussion” section for more details on this subject).

The ability of SARS-CoV-2 mAbs to select any of these variants that is apparently fit and that naturally occurs even at low frequencies in circulating viral populations suggests that the therapeutic use of single mAb might select for escape mutants, although the extent to which resistance will impact the effectiveness of Abs in SARS-CoV-2 therapeutic and vaccine settings is still a matter of intense investigation^10,18,53,54^. In this arena, the purpose of this work is to provide an atomistic-based, *in silico* perspective of the role eventually played by currently circulating S-RBD_CoV‑2_ mutations in escaping binding of the two mAbs bamlanivimab and etesevimab as a proof of concept. A computational alanine scanning (CAS) mutagenesis^55^ initially allowed us to identify the main molecular determinants of each Ab/S-RBD_CoV‑2_ recognition; then, each spike residues that, according to the CAS results, contributes to the relevant viral protein/mAb binding interface was mutated into all currently reported circulating mutations at that position^56^, and the corresponding variation in affinity of all mutated spikes for each mAb with respect to the wild-type viral protein was estimated using a consolidate protocol^57^. To quickly and effectively ranking the different spike mutants with respect to their mAb escaping potential, a color-coded criterion based on the predicted free energy difference range of values was adopted, as shown in Table 1. Of note, this criterion is identical to the one we adopted and validated in our previous work for ranking the effect of both ACE2 and S-RBD_CoV‑2_ mutations on their mutual binding^57^.

**Table 1:** Color-coded criterion based on the predicted free energy difference (ΔΔG) range of values adopted to rank the affinity of SARS-CoV-2 spike mutants for the LY-CoV-555 (bamlanivimab) and LY-CoV016 (etesevimab) monoclonal antibodies. Negative/positive ΔΔG values indicate unfavorable/favorable substitutions for the mutant residue in the relevant position, respectively.

Mutation effect	ΔΔG range (kcal/mol)	Color code
Neutral mutations	− 0.25 ≤ ΔΔG ≤ + 0.25	Gray
Mildly destabilizing mutations	− 2.00 ≤ ΔΔG < − 0.25	Light yellow
Destabilizing mutations	− 4.00 ≤ ΔΔG < − 2.00	Light red
Highly destabilizing mutations	− 4.00 < ΔΔG	Red

## Results

### Computational alanine scanning of the SARS-CoV-2 spike protein residues at the binding interface with the LY-CoV555 (bamlanivimab) monoclonal antibody

**Figure 2:**
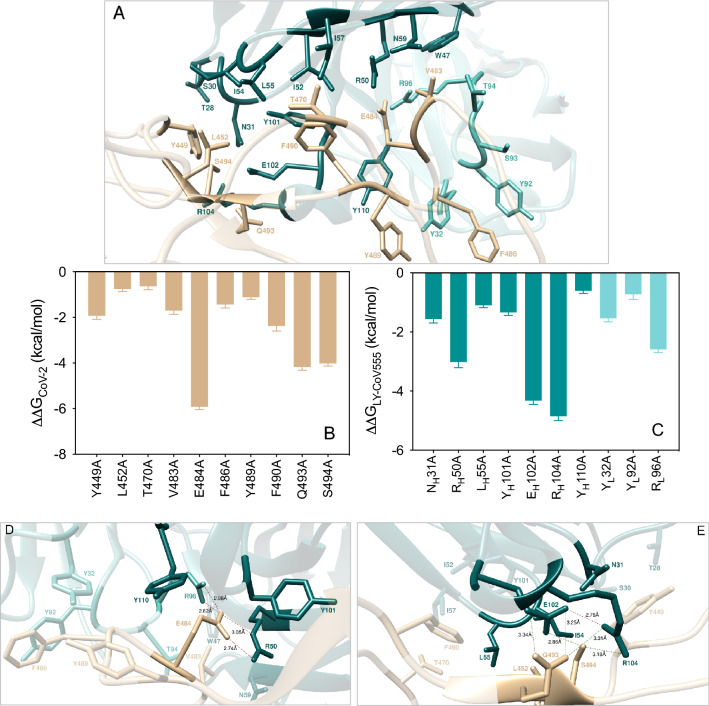
(**A**) Structural details of the binding interface between the LY-CoV555 (bamlanivimab) mAb and the viral spike protein receptor-binding domain of SARS-CoV-2 (S-RBD_CoV‑2_). The secondary structures of the S-RBD_CoV‑2_ is shown as a light tan ribbon, while the HC and LC of the LY-CoV555 mAb are portrayed as light teal and light Tiffany ribbons, respectively. Each interacting protein residue is highlighted in dark matching-colored sticks and labeled. Binding energy change (ΔΔG = ΔG_WILD-TYPE _– ΔG_ALA_) obtained from the computational alanine-scanning (CAS) mutagenesis for the S-RBD_CoV‑2_ residues at the binding interface with the LY-CoV555 mAb (**B**) and for the LY-CoV555 mAb residues at the binding interface with the viral protein RBD (**C**). Negative ΔΔG values indicate unfavorable substitution for alanine in the relevant position. For the numerical values of ΔΔG and all related energy terms, see the text and Tables S2 and S3. Main interactions involving the viral S-RBD_CoV‑2_ residues E484 (**D**), and Q493, S494 and L452 (**E**) at the interface with the LY-CoV555 mAb as obtained from equilibrated MD simulations. Each S-RBD_CoV‑2_ residue under discussion and all other residues directly interacting with it are highlighted in dark matching-colored sticks and labeled; further residues/interactions related to the residue under investigation are evidenced in light matching-colored sticks and labelled in light gray. Hydrogen bonds (HBs) and salt bridges (SBs) are represented as dark green and dark red broken lines, respectively, and the relevant average distances are reported accordingly. Further important HBs and SBs detected in each complex are also indicated using light green/red broken lines and light gray labels (see Table S1 for details).

Within distance and energetic cutoffs of 4.0 Å and 0.5 kcal/mol, respectively, the analysis of the equilibrated molecular dynamics (MD) trajectory of LY-CoV555 in complex with the S-protein RBD of SARS-CoV-2 shows that a total of 10 residues of the S-RBD_CoV‑2_ stably and effectively contact 19 residues of the fragment antigen-binding (Fab) portion of the LY-CoV555 mAb, 14 of which locate on the heavy chain (HC) and 5 on the light chain (LC), respectively (Fig. 2A and Table S1).

The results from the CAS (Fig. 2B,C) identify both the S protein and the LY-CoV555 mAb residues that afford a significant contribution to the binding interface. Furthermore, CAS data clearly indicate residues E484, Q493 and S494 on the S-RBD_CoV‑2_ and R_H_50, R_H_96, E_H_102, and R_H_104 on the mAb HC as key positions contributing to shaping and determining the stability of the relevant protein–protein interface, as discussed in details below.

#### E484

The confirmation of the E484 as a crucial residue was an expected result as a glutamic acid (E) to lysine (K) substitution at this position (E484K) in the S-RBD_CoV‑2_ is present in the rapidly spreading variants of concern belonging to the B.1.351 (aka South African and now Beta, according to the new WHO labeling system) and P.1 (Brazilian or Gamma) lineages, while the E484Q/L452R double mutation is a component of the B.1.617 (Delta/Kappa) lineage that is currently dramatically spreading in India (vide infra). E484 locates at the tip of a long, flexible loop in the S-RBD_CoV‑2_; as such, any intermolecular interaction involving E484 and LY-CoV555 could be important in eventually anchoring the entire superstructure. The MD trajectory of the S-RBD_CoV‑2_/LY-CoV555 complex shows that E484 is involved in two tight and bifurcated salt-bridges with residues R_H_50 (2.74 ± 0.10 Å and 3.05 ± 0.14 Å) and R_L_96 (2.82 ± 0.11 Å and 2.96 ± 0.13 Å) on the mAb HC and LC, respectively, flanked by contact interactions (CIs) with the side chains of Y_H_110 and Y_H_101 (Fig. 2D, Table S1). When E484 is replaced with alanine in CAS, these interface-stabilizing interactions—along with the slightly beneficial contribution from the intramolecular van der Waals contacts with the two Ab HC tyrosines—are no longer made, reflecting a loss of the corresponding binding free energy of ΔΔG_CoV-2_(E484A) = − 5.92 ± 0.12 kcal/mol (Fig. 2B, Table S2). Contextually, the corresponding values of ΔΔG_LY-CoV555_(R_H_50A) = − 3.02 ± 0.19 kcal/mol and ΔΔG_LY-CoV555_(R_L_96A) = − 2.59 ± 0.11 kcal/mol (Fig. 2C, Table S3) are in line with the important contribution these residues provide to the formation of the corresponding viral protein/antibody interface described above (Fig. 2D).

#### Q493

At the 493 position of the SARS-CoV-2 S protein, Q493 forms three stabilizing HBs across the protein–protein interface, one with the side chain of LY-CoV555 E_H_102 (3.25 ± 0.18 Å) and two with the side chain and the C=O backbone of R_H_104 (3.31 ± 0.12 Å and 3.34 ± 0.17 Å), respectively (Fig. 2E, Table S1). Thus, abrogation of these intermolecular contacts by replacing the wild-type glutamine with alanine is accompanied by a ~ 4.2 kcal/mol loss in binding free energy (ΔΔG_CoV-2_(Q493A) = − 4.18 ± 0.14 kcal/mol, Fig. 2B, Table S2). Similarly, when either of the two LY-CoV555 residues E_H_102 or R_H_104 are mutated into alanine, the related values of ΔΔG nicely reflect their importance in binding S-RBD_CoV‑2_, as ΔΔG_LY-CoV555_(E_H_102A) = − 4.32 ± 0.13 kcal/mol and ΔΔG_LY-CoV555_(R_H_104A) = − 4.58 ± 0.15 kcal/mol, respectively (Fig. 2C, Table S3). Of note, these two mAb amino acids are engaged in a fundamental internal SB (2.76 ± 0.11 Å, Fig. 2E, Table S1) that appears to play a major structural role in properly orienting their side chains for binding both Q493 and another spike key residue—S494—as discussed below.

#### S494

Serine 494 is an interesting SARS-CoV-2 RBD residue that has been previously reported by us to form an internal HB with the side chain of the adjacent Q493, instrumental to direct the latter in H-bridging aspartic acid 35 (D35) on the human ACE2 across their binding interface^55,57^. When in complex with LY-CoV555, S494 engages the side chains of the mAb residues E_H_102 and R_H_104 in two intermolecular HBs (2.86 ± 0.16 Å and 3.18 ± 0.19 Å, respectively), alongside a strong polar interaction with the viral N31 (Fig. 2E, Table S1). Thus, the S494A mutation actually shows a considerable variation in the corresponding ΔΔG value (ΔΔG_CoV‑2_(S494A) = − 4.02 ± 0.12 kcal/mol, Fig. 2B, Table S2), making S494 the spike third protein/protein key residue.

#### V483, F486, and Y489

Predictably, the SARS-CoV-2 RBD residues V483, F486, and Y489 afford only a network of stabilizing intermolecular CIs to the viral protein/antibody binding interface region centered around the nearby key residue E484 (Fig. 2D). Specifically, the side chain of V483 interacts—via van der Waals/hydrophobic contacts—with the side chains of W_H_47, R_H_50 and N_H_59 on the Ab HC, and the side chains of T_L_94 and R_L_96 on the Ab LC, respectively (Fig. 2D, Table S1). Contextually, the phenyl ring of F486 engages two π/π stacking interactions involving the side chains of the Ab LC Y_L_32 and Y_L_92, while the aromatic moiety of Y489 establish dispersive/polar interactions with the LY-CoV555 residues Y_H_110 and Y_L_32 on the Ab HC and LC, respectively (Fig. 2D, Table S1). The absence of these CIs when each of these residues is mutated into alanine reflects the moderate variations of the corresponding free energy of binding (Fig. 2B, Table S2), that is, ΔΔG_CoV‑2_(V483A) = − 1.70 ± 0.18 kcal/mol, ΔΔG_CoV‑2_(F486A) =  − 1.44 ± 0.15 kcal/mol, and ΔΔGG_CoV‑2_(Y489A) = − 1.12 ± 0.09 kcal/mol, respectively.

#### Y449, L452, T470, and F490

In analogy to what just discussed a few lines above, the main role of residues Y449, L452, T470 and F490 on the S-RBD_CoV‑2_ is also to reinforce the viral protein/antibody binding interface region centered—in this case—around the two other important residues Q493 and S494 by providing a number of favorable intermolecular CIs (Fig. 2E). In particular, Y449 provides three stabilizing polar interactions with the side chains of T_H_28, S_H_30, and N_H_31, and is in van der Waals distance with I_H_54, all on the Ab HC (Fig. 2E, Table S1). L452 contacts the side chains of I_H_54 and L_H_55 on the LY-Cov555 Ab HC, while the last two spike residues T470 and F490 exchange nonpolar interactions with the side chains of the Ab HC residues I_H_52, I_H_54, L_H_55 and I_H_57, in addition to the π/π stacking observed between F490 and the Ab HC Y_H_101 (Fig. 2E, Table S1). This is supported by the calculated ΔΔG value obtained by changing these amino acids into alanine in the S-RBD_CoV‑2_/LY-CoV555 Ab complex, that is, ΔΔG _CoV‑2_(Y449A) = − 1.93 ± 0.16, ΔΔG_CoV‑2_(L452A) = − 0.76 ± 0.11 kcal/mol, ΔΔG_CoV‑2_(T470A) = − 0.64 ± 0.15, and ΔΔG_CoV‑2_(F490A) = − 2.38 ± 0.22 (Fig. 2B, Table S2).

### In silico mutagenesis of the SARS-CoV-2 spike protein residues at the binding interface with the LY-CoV555 (bamlanivimab) monoclonal antibody

The recent survey of data reported by Starr et al.^56^ led to the following list of naturally occurring mutations at the SARS-CoV-2 spike protein residues contacting the LY-CoV555 mAb: E484A/D/G/K/Q/R/V, Q493H/K/L/R, S494A/P/R/T, L452M/Q/R, Y449D/F/H/N/S, T470A/I/K/N, V483A/F/G/I/L, F486I/L/S, Y489C/F/H/S, and F490L/S/V/Y. In what follows, we report and discuss the different effects exerted by each of these spike mutant residues on the structure and strength of the resulting S-RBD_CoV‑2_/LY-CoV555 binding interface. In analogy with our previous work focused on the estimation of the difference in binding affinity between different allelic variants of ACE2 or S-RBD_CoV‑2_^57^, in this study we adopted the same color-coded criterion based on the predicted free energy difference range of values shown in Table 1.

#### E484

**Figure 3:**
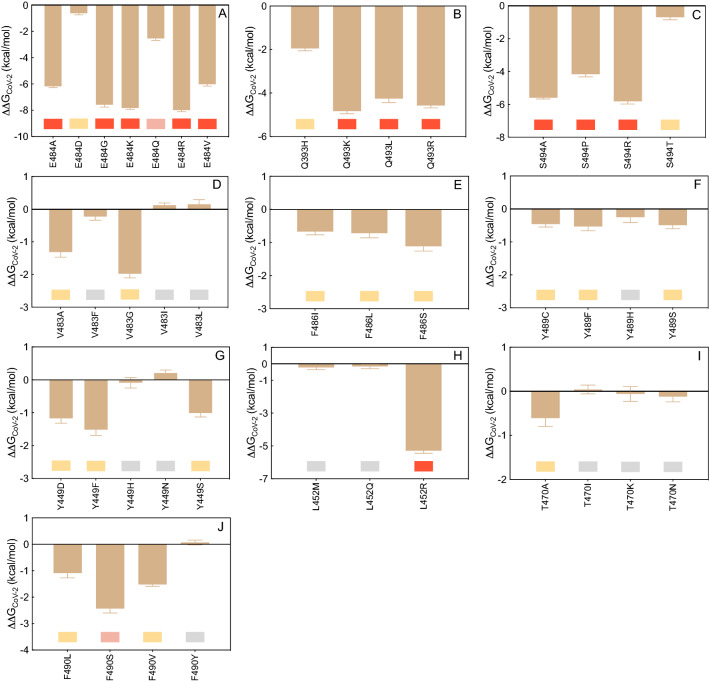
Change in binding free energy (ΔΔG = ΔG_WILD-TYPE_ − ΔG_MUTANT_) predicted by computational mutagenesis of the S-RBD_CoV‑2_ wild-type residues E484 (**A**), Q493 (**B**), S494P (**C**), V483 (**D**), F486 (**E**), Y489 (**F**), Y449 (**G**), L452 (**H**), T470 (**I**), and F490 (**J**) for the corresponding S-RBD_CoV‑2_/LY-CoV555 mAb complexes. Negative ΔΔG values indicate unfavorable substitutions for the mutant residue in the relevant position. In this and all other similar figures, the colored boxes below each bar in the graphs show the classification of the destabilizing effects of the corresponding mutation on the S-RBD_CoV‑2_/LY-CoV555 mAb complex. Color legend: gray, neutral mutations; light yellow, mildly destabilizing mutations; light red, destabilizing mutations; red, highly destabilizing mutations (see Table 1). The numerical values of ΔΔG, all related energy terms, and all underlying intermolecular intramolecular interactions are reported in Tables S4–S15 and Figures S1–S10.

**Figure 4:**
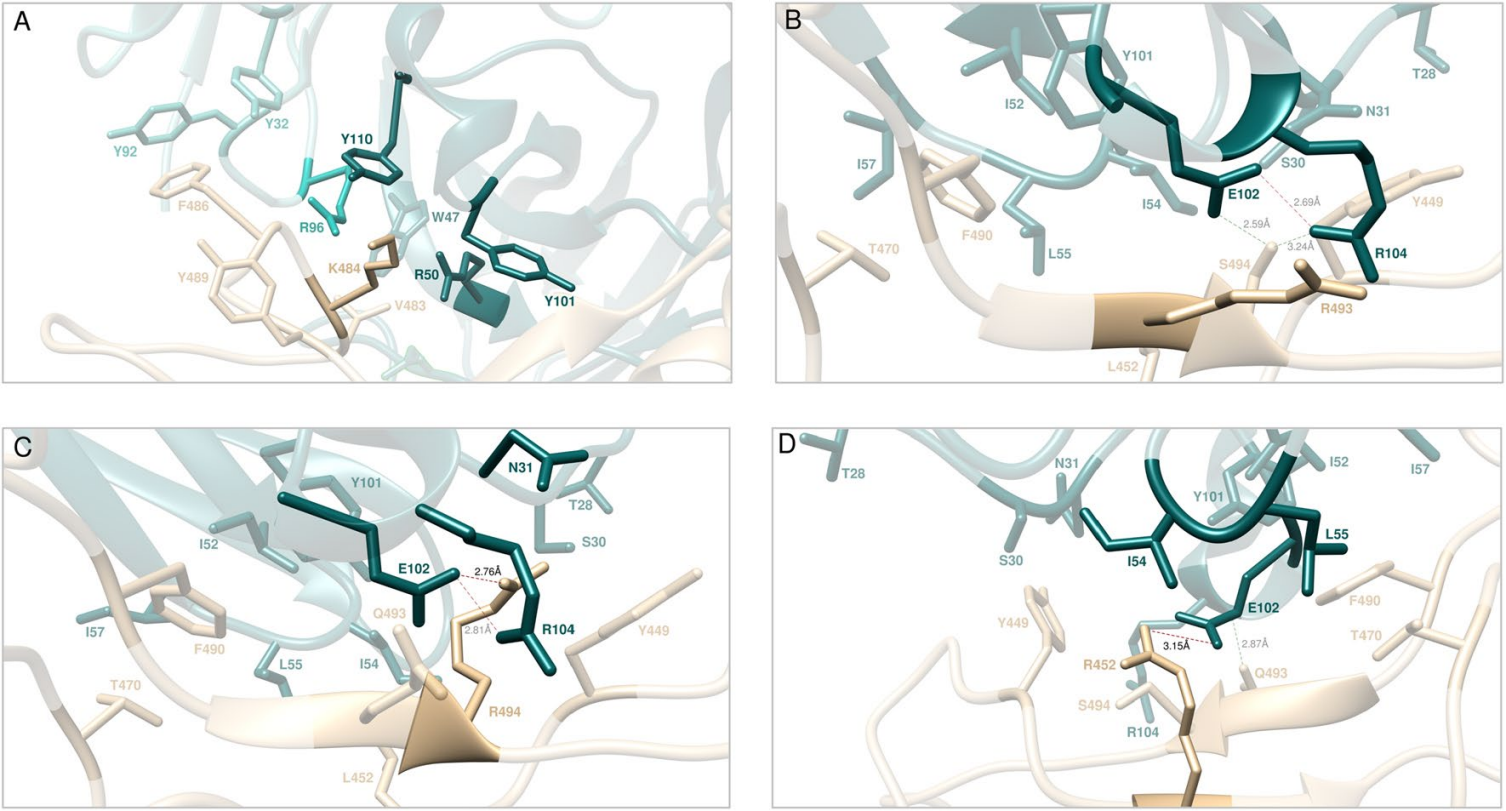
Main interactions involving the S-RBD_CoV‑2_ E484K (**A**), Q3493R (**B**), S494R (**C**), and L452R (**D**) mutants at the interface with the LY-CoV555 (bamlanivimab) mAb as obtained from the relevant equilibrated MD simulations. Images for all other circulating mutants (E484A/D/G/Q/V, Q493H/K/L, S494A/P/T, and L452M/Q) are shown in Figures S1–S3 and S8 (see also Tables S5–S7 and S12 for details). Colors and other explanations as in Fig. 2.

The CAS results discussed above clearly show that glutamic acid at the position 484 along the S protein wild-type sequence (E484) is a key player in the LY-CoV555/S-RBD_CoV‑2_ interaction (Fig. 2B,D, Tables S1, S2). Interesting, replacing the viral spike E484 with each of the alternative residues considered (i.e., E484A/D/G/K/Q/R/V) reflects into a robust interface disrupting behavior, with the mild exception of the E484D substitution (Fig. 3A, Figure S1, and Tables S4, S5). Figure 4A shows the results for the E484K as a representative example. As seen from this Figure, in the presence of the K484 mutation the two topical, bifurcated interface-stabilizing SBs between E484 and the side chains of LY-CoV55 R_H_50 and R_L_96 (Fig. 2D, Table S5) cannot obviously be established, while the background network of CIs involving residues V483, F486, and Y489 on the S protein and W_H_47, Y_L_92, Y_H_110 and Y_H_32 on the LY-CoV555 mAb remains almost unperturbed (Fig. 4A, Figure S1, Table S5). An utterly similar situation is observed in the presence of the A, G, R, and V mutants (see Figure S1 and Table S5 for details). In line with this, the predicted changes in binding free energy for the replacement of the wild-type E484 with A/G/K/R/V in the S-RBD_CoV‑2_/LY-CoV555 relevant complexes (ΔΔG_CoV‑2_(E484A) = − 6.18 ± 0.10 kcal/mol, ΔΔG_CoV‑2_(E484G) = − 7.58 ± 0.18 kcal/mol, ΔΔG_CoV‑2_(E484K) =  − 7.83 ± 0.11 kcal/mol, ΔΔG_CoV‑2_(E484R) = − 7.99 ± 0.12 kcal/mol, ΔΔG_CoV‑2_(E484V) = − 6.02 ± 0.14 kcal/mol, Fig. 3A and Table S4) support the prominent contribution played by this residue in anchoring the viral protein/LY-CoV555 mAb binding interface and the LY-CoV555 escaping potential of the E484A, E484G, E484K, E484R, and E484V SARS-CoV-2 circulating mutants. The charged-to-neutral isosteric replacement E484Q has a moderately destabilizing effect (ΔΔG_CoV‑2_(E484Q) = − 2.53 ± 0.16 kcal/mol, Fig. 3A, Table S4), since the two strong bifurcated SBs characterizing the wild-type complex are replaced with two single HBs, one between the side chain of Q484 and the side chain of arginine at position 50 of the mAb HC (2.96 ± 0.11 Å), and one between the backbone C=O group of Q484 and the side chain of arginine 96 of the Ab LC (2.70 ± 0.16 Å), respectively (Figure S1, Table S5). Finally, similarly to E484 the mutated D484 can establish SB interactions with the side chains of LY-CoV555 R_H_50 (2.94 ± 0.13 Å) and R_L_96 (2.81 ± 0.19 Å and 3.26 ± 0.22 Å), along with the full network of CIs seen in the wild-type complex, overall resulting in a predicted neutral effect on the related protein/protein interface (ΔΔG_CoV‑2_(E484D) = − 0.61 ± 0.13 kcal/mol, Fig. 3A, Table S4, Figure S1 and Table S5).

#### Q493

According to relevant CAS-based prediction, Q493 also plays a primary stabilizing role at the S-protein/LY-CoV555 mAb interface (Fig. 2B, Tables S1, S2). The analysis of the MD trajectories of all considered mutants (Q493H/K/L/R) reveals that, with respect to the wild-type Q493, all residues except H493 induce a strong destabilizing effect at the interface with the LY-CoV555 mAb (Fig. 3B, Figure S2, Tables S4 and S6). With R493 as a proof-of-principle, Fig. 4B shows that this mutant is no longer able to form the three fundamental HBs across the protein/protein interface with E_H_102, and R_H_104 on the Ab heavy chain, respectively (see also Table S6). Moreover, the spike Y449 no longer engages the side chains of the two LY-CoV555 mAb HC residues T_H_28 and S_H_31 in polar interactions, leaving the rest of the CI network substantially unchanged (Figs. 2E and 4B, Table S6). Accordingly, the predicted affinity of this mutant viral protein for the LY-CoV55 mAb is markedly lower than that of the native counterpart (ΔΔG_CoV‑2_(Q493R) = − 4.57 ± 0.11 kcal/mol, Fig. 3B, Table S4). Analogous effects are predicted for the other two mutants Q493K and Q493L, reflecting in a comparable decrease of protein/protein binding strength (ΔΔG_CoV‑2_(Q493K) = − 4.83 ± 0.12 kcal/mol and ΔΔG_CoV‑2_(Q493L) = − 4.26 ± 0.18 kcal/mol, respectively, Fig. 3B, Table S4, Figure S2 and Table S6). These data therefore suggest that the Q493K/L/R mutants could all be LY-CoV555 escaping mutants. At variance with these, mutating Q493 into histidine introduce a somewhat less drastic changes in the topology of the viral protein–antibody interface. In particular, Q493H is still able to preserve one HB with the side chain of the LY-CoV555 R_H_104 (3.39 ± 0.15 Å) while the second HB interaction with the same mAb residue is replaced by a π/cation interaction (Figure S2, Table S6). Notably, the HB between H493 and E_H_102 is also missing in the entire MD trajectory of this S-RBD_CoV‑2_ mutant/mAb complex, in addition to the polar CI between Y449 on spike and T_H_28 on LY-CoV555 (Figure S2, Table S6). In line with this, a moderate variation of the corresponding free energy of binding (Fig. 3B, Table S4), that is, ΔΔG_CoV‑2_(Q493H) = − 1.95 ± 0.11 kcal/mol.

#### S494

The third SARS-CoV-2 spike position highlighted by the CAS results as a key residue in binding the LY-CoV555 mAb is S494 (Fig. 2B). Mutagenesis of this residue into A, P, R, and T reflects into strong interface destabilizing effects, exception made for the S494T substitution for which only a mild effect is observed (Fig. 3C, Table S4). In the case of the R494 mutant, the current MD simulations show that both main intermolecular HBs in which the wild-type residue is involved (i.e., S494-E_H_102 and S494-R_H_104) are no longer detected in the trajectory of the correspondent mutant viral protein/mAb complex (Fig. 4C, Table S7). Also, two out of three further stabilizing HBs between the adjacent and important Q493 residue on S-RBD_CoV‑2_ and the side chains of LY-CoV555 E_H_102 and R_H_104 are no longer formed in the presence of the R494 mutation (Figs. 2E and 4C, Table S7). These evidences, along with several missing stabilizing CIs at the protein/protein interface (see Table S7 for details), concur to lower the predicted affinity of the R494 mutant S-RBD_CoV‑2_ for the LY-CoV555 mAb (ΔΔG_CoV‑2_(S494R) = − 5.81 ± 0.17 kcal/mol, Fig. 3C, Table S4). Accordingly, the three circulating mutants S494A, S494P, and S494R are all predicted to be potential LY-CoV555 escaping variants. When S-RBD_CoV‑2_ S494 is mutated into threonine (S494T), the MD-predicted interaction network at the corresponding Ab binding interface is only moderately perturbed with respect to that described above for the wild-type complex; in particular, only the HBs between T494 on the SARS-CoV-2 RBD and E_H_102 on the HC of LY-CoV555, and between the viral Q493 and the same glutamic acid on the mAb HC are replaced by two polar CIs (Figure S3, Table S7). In line with this, the related value of ΔΔG_CoV‑2_(S494T) is slightly unfavorable and equal to -0.70 ± 0.15 kcal/mol (Fig. 3C and Table S4).

#### V483, F486, and Y489

The mutagenesis results obtained by mutating these three viral spike amino acids into the reported variants (V483A/F/G/I/L, F486I/L/S, and Y489C/F/H/S, respectively) ultimately confirm the minor role played by these residues at the SARS-CoV-2 RBD/LY-CoV555 mAb binding interface (Fig. 3D–F, Table S4). Indeed, the analysis of the respective MD trajectories reveals that each interaction network is practically conserved in all relevant supramolecular assemblies (see Figures S4–S6 and Tables S8–S10 for details). Accordingly, the SARS-CoV-2 spike mutations at residues 483, 486, and 489 reported so far in circulating viral populations are predicted to be tolerated at each respective position.

#### Y449, L452, T470 and F490

As it could be anticipated from the relevant CAS data discussed above, the in silico mutagenesis results for these further four viral protein residues into the reported variants (Y449D/F/H/N/S, L452M/Q/R, T470A/I/K/N and F490L/S/V/Y) also confirm a remarkable degree of tolerability to substitution at each of these spike positions in binding the LY-CoV555 Ab, with the remarkable exceptions of the L452R and—albeit to a lower extent—the F490S mutations (Fig. 3G–J, Table S4, Figures S7–S10, Tables S11–S14). Together with T470 and F490, the spike residue L452 is a part of a hydrophobic region at the binding interface with LY-CoV555; accordingly, when this amino acid is replaced by the small non-polar methionine or even by the polar glutamine, the corresponding viral/antibody interface remains almost unaffected (Fig. 3H and Fig. S8, Table S12). On the contrary, upon mutation of L452 into the positively charged and long-chained asparagine, a substantial modification of the relative binding region is observed in the corresponding MD trajectory, as shown in Fig. 4D. Specifically, the structurally-important internal SB between the side chains of LY-CoV555 E_H_102 and R_H_104 on the Ab HC (Fig. 2E) is no longer detected, as it is replaced by an analogous yet intermolecular interaction between the former mAb residue and the mutant spike R452 (3.15 ± 0.11 Å). Contextually, however, the formation of this new interface SB is accompanied by the loss of all other crucial intermolecular interactions involving both S-RBD_CoV‑2_ key residues Q493 and S494 (Fig. 4D, Table S12). This, in turn, properly reflects in the substantial variation of the corresponding binding free energy value, so that ΔΔG_CoV‑2_(L452R) = − 5.29 ± 0.15 kcal/mol, Fig. 3H, Table S4), thereby supporting the LY-CoV555 escaping potential of this SARS-CoV-2 spike isoform.

### Computational alanine scanning of the SARS-CoV-2 spike protein residues at the binding interface with the LY-CoV016 (etesevimab) monoclonal antibody

**Figure 5:**
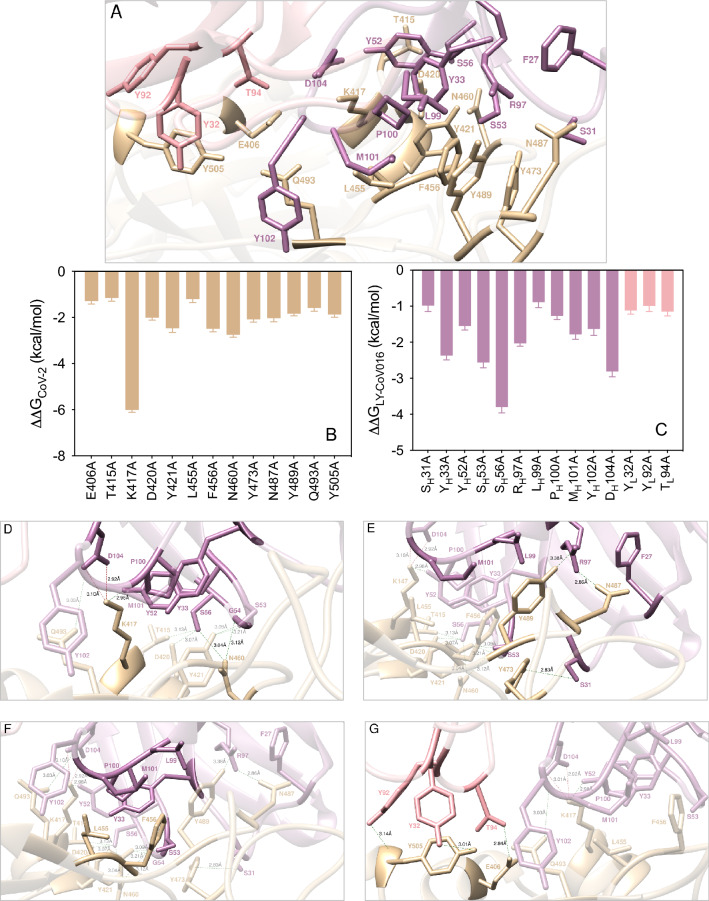
(**A**) Structural details of the binding interface between the LY-CoV016 (etesevimab) mAb and the viral spike protein receptor-binding domain of SARS-CoV-2 (S-RBD_CoV‑2_). The secondary structures of the S-RBD_CoV‑2_ is shown as a light tan ribbon, while the secondary structures of the HC and LC of the LY-CoV016 mAb are portrayed as light mulberry and light pink icing ribbons, respectively. Each interacting protein residue is highlighted in dark matching-colored sticks and labeled. Binding energy change (ΔΔG = ΔG_WILD-TYPE _– ΔG_ALA_) obtained from the computational alanine-scanning (CAS) mutagenesis for the S-RBD_CoV‑2_ residues at the binding interface with the LY-CoV016 mAb (**B**) and for the LY-CoV016 mAb residues at the binding interface with the viral protein RBD (**C**). Negative ΔΔG values indicate unfavorable substitution for alanine in the relevant position. For the numerical values of ΔΔG and all related energy terms, see the text and Tables S16 and S17. Main interactions involving the viral S-RBD_CoV‑2_ residues K417 and N460 (**D**), Y473, N487, and Y489 (**E**), L455 and F456 (**F**), and E406 and Y505 (**G**) at the interface with the LY-CoV016 mAb as obtained from equilibrated MD simulations. Each S-RBD_CoV‑2_ residue under discussion and all other residues directly interacting with it are highlighted in dark matching-colored sticks and labeled; further residues/interactions related to the residue under investigation are evidenced in light matching-colored sticks and labelled in light gray. Hydrogen bonds (HBs) and salt bridges (SBs) are represented as dark green and dark red broken lines, respectively, and the relevant average distances are reported accordingly. Further important HBs and SBs detected in each complex are also indicated using light green/red broken lines and light gray labels (see Table S1 for details).

Within the same distance and energetic cutoffs adopted for the analysis of the viral S-protein/LY-CoV555 mAb complex (4.0 Å and 0.5 kcal/mol, respectively), the inspection of the equilibrated MD trajectory of the alternative S-RBD_CoV‑2_/LY-CoV016 mAb assembly reveals that 13 viral protein residues persistently contact 16 residues of the Fab portion of the LY-CoV016 Ab at the relative binding interface. Of the latter, 13 amino acids locate on the mAb HC and 3 belong to the mAb LC, respectively (Fig. 5A, Table S15). According to the CAS results shown in Fig. 5B,C (see also Tables S16, S17), and at variance with the binding mode just discussed for the alternative mAb LY-Cov555, the viral RBD/LY-CoV016 binding interface is substantially more diffused and characterized by four distinct regions, the first of which locates in the area centered around the S-RBD_CoV‑2_ residues K417 and N460 (Fig. 5D). Specifically, the MD trajectory of the S-RBD_CoV‑2_/LY-CoV016 complex shows that K417 binds the side chain of D_H_104 on the mAb HC via a bifurcated SB (2.92 ± 0.15 Å and 3.10 ± 0.17 Å). Additionally, the K417 side chain is also involved in a stable HB with Y_H_52 (2.98 ± 0.14 Å) and in CI distance with the side chains of Y_H_33 (ΔΔG_LY-CoV016_(Y_H_33A) = − 2.37 ± 0.12 kcal/mol) and P_H_100 (ΔΔG_LY-CoV016_(P_H_100A) = − 1.27 ± 0.10 kcal/mol, see Fig. 5C,D and Tables S15 and S17). In agreement with this interaction pattern, the K417A mutation in CAS reduces the binding affinity of the corresponding S-RBD_CoV‑2_ for the LY-CoV016 mAb by 6 kcal/mol (ΔΔG_CoV‑2_(K417A) = − 6.01 ± 0.10 kcal/mol, Fig. 5B, Table S16). In the same context, the corresponding values of ΔΔG_LY-CoV016_(D_H_104A) = − 2.81 ± 0.15 kcal/mol and ΔΔG_LY-CoV016_(Y_H_52A) = − 1.55 ± 0.11 kcal/mol (Fig. 5C, Table S17) properly rank the relative importance of these LY-Cov016 mAb residues at corresponding viral protein/antibody interface described above. On the other hand, the S-RBD_CoV‑2_ N460 residue is involved in two permanent HBs with the side chain of S_H_56 (3.04 ± 0.09 Å) and with the oxygen atom of the backbone of G_H_54 (3.12 ± 0.17 Å), and the relevant value of ΔΔG obtained by CAS for the N460A mutation (ΔΔG_CoV-2_(N460A) = − 2.75 ± 0.11 kcal/mol, Fig. 5B, Table S16) confirms the fundamental role of this spike residue at the binding interface. Interestingly, the change in binding free energy predicted by CAS for the S_H_56A substitution on the LY-Cov016 mAb (ΔΔG_LY-CoV016_(S_H_56A) = − 3.80 ± 0.16 kcal/mol, Fig. 5C, Table S17) accounts for the existence of additional stabilizing interactions in this region, characterized by a strong and virtuous network of HBs and CIs. In detail, S_H_56 is also involved in two stable HBs with the hydroxyl group of T415 (3.13 ± 0.10 Å, ΔΔG_CoV-2_(T415A) = − 1.16 ± 0.14 kcal/mol) and the side chain of D420 (3.07 ± 0.15 Å, ΔΔG_CoV-2_(D420A) = − 2.01 ± 0.11 kcal/mol), respectively (Fig. 5B,D, Tables S15, S16). Moreover, two additional S-RBD_CoV‑2_ residues concur in determining the stability of the protein/protein interface (Fig. 5D, Table S15): Y421 (ΔΔG_CoV-2_(Y421A) = − 2.47 ± 0.18 kcal/mol) and Q493 (ΔΔG_CoV-2_(Q493A) = − 1.59 ± 0.14 kcal/mol) (Fig. 5B and Table S16). Specifically, the spike tyrosine 421 performs two HBs with LY-CoV016 S_H_53 (3.09 ± 0.11 Å, ΔΔG_LY-CoV016_(S_H_53A) = − 2.56 ± 0.15 kcal/mol) and the nitrogen backbone atom of G_H_54 (3.21 ± 0.14 Å), respectively, while glutamine 493 is involved in the same type of intermolecular interaction with the amide moiety of the backbone of Y_H_102 (3.03 ± 0.18 Å), in addition to a favorable CI with the side chain of M_H_101 (Fig. 5C,D, Tables S15, S17).

The second important region for the stabilization of the S-RBD_CoV‑2_/LY-CoV016 complex is mainly composed by the viral residues Y473, N487 and Y489 (Fig. 5E, Table S15) and the mAb HC residue R_H_97. Indeed, two HBs are detected between the guanidine moiety of R_H_97 (ΔΔG_LY-CoV016_(R_H_97A) = − 2.03 ± 0.08 kcal/mol, Fig. 5C, Table S17) and the side chain of N487 (2.86 ± 0.15 Å, ΔΔG_CoV-2_(N487A) = − 2.03 ± 0.16 kcal/mol) and the hydroxyl group of Y489 (3.38 ± 0.13 Å, ΔΔG_CoV-2_(Y489A) = − 1.84 ± 0.09 kcal/mol), respectively (Fig. 5B,E, and Tables S15, S16). Additionally, the CIs of these viral protein amino acids with the LY-CoV016 HC residues F_H_27, L_H_99 (ΔΔG_LY-CoV016_(L_H_99A) = − 0.89 ± 0.15 kcal/mol), and M_H_101 (ΔΔG_LY-CoV016_(M_H_101A) = − 1.78 ± 0.14 kcal/mol) further contribute to binding interface stabilization (Fig. 5E, Table S15, Fig. 5C, Table S17). Moreover, the S-RBD_CoV‑2_ Y473 (ΔΔG_CoV-2_(Y473A) = − 2.08 ± 0.12 kcal/mol) is stably engaged in an HB with the side chain of S_H_31 (2.83 ± 0.21 Å, ΔΔG_LY-CoV016_(S_H_31A) = − 0.98 ± 0.17 kcal/mol) and in a polar interaction with S_H_53 (Fig. 5B,C,E, Tables S15–S17).

Located in between the two protein/protein interface regions just described, the third binding zone is identified by a network of van der Waals and hydrophobic interactions mainly involving the S-RBD_CoV‑2_ residues L455 (ΔΔG_CoV-2_(L455A) = − 1.20 ± 0.16 kcal/mol) and F456 (ΔΔG_CoV-2_(F456A) = − 2.49 ± 0.13 kcal/mol) (see Fig. 5B,F, Tables S1 and S16). In particular, this hydrophobic patch at the S-RBD_CoV‑2_/LY-CoV016 mAb interface sees F456 as the pivot point that coordinates and to appropriately orient the mAb residues Y_H_33, S_H_53, L_H_99, P_H_100 and M_H_101 for further protein/protein interactions (Fig. 5F, Table S15).

Finally, the last detected binding region—although apparently not a primary determinant of the viral/Ab interface stabilization—supports the optimization of the mutual protein/protein recognition. Indeed, this region involves only the LY-CoV016 mAb LC residues Y_L_32 (ΔΔG_LY-CoV016_(Y_L_32A) = − 1.12 ± 0.10 kcal/mol), Y_L_92 (ΔΔG_LY-CoV016_(Y_L_92A) = − 0.99 ± 0.16 kcal/mol), and T_L_94 (ΔΔG_LY-CoV016_(T_L_94A) = − 1.15 ± 0.12 kcal/mol) in a set of stable HBs with the viral spike residues E406 (2.84 ± 0.27 Å, ΔΔG_CoV-2_(E406A) = − 1.29 ± 0.13 kcal/mol), the hydroxyl group of Y505 (3.01 ± 0.19 Å, ΔΔG_CoV-2_(Y505A) = − 1.87 ± 0.12 kcal/mol), and the nitrogen backbone atom of the same tyrosine (3.14 ± 0.13 Å) (Fig. 5B,C,G, and Tables S15–S17).

### In silico mutagenesis of the SARS-CoV-2 spike protein residues at the binding interface with the LY-CoV555 (etesevimab) monoclonal antibody

The same data survey reported by Starr and coworkers^56^ led us to identify the following naturally occurring mutations at the SARS-CoV-2 spike protein residues contacting the LY-CoV016 Ab: E406D/Q, T415A/I/N/P/S, K417E/N/R/T, D420A/G/N, L455F/S/V, F456L/Y, N460I/K/S/T, Y473F/H, N487D, Y489C/F/H/S, Q493H/K/L/R and Y505F/H/W. Below, we report and discuss different effects exerted by each of these spike mutant residues on the structure and strength of the resulting S-RBD_CoV‑2_/LY-CoV016 binding interface by adopting again the same color-coded criterion shown in Table 1.

#### K417

**Figure 6:**
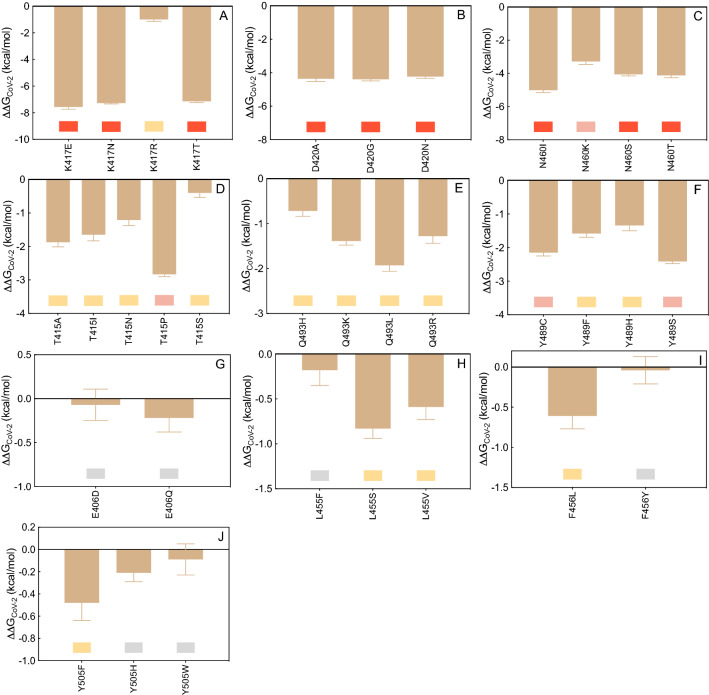
Change in binding free energy (ΔΔG = ΔG_WILD-TYPE_ − ΔG_MUTANT_) predicted by computational mutagenesis of the S-RBD_CoV‑2_ wild-type residues K417 (**A**), D420 (**B**), N460 (**C**), T415 (**D**), Q493 (**E**), Y489 (**F**), E406 (**G**), L455 (**H**), F456 (**I**), and Y505 (**J**) for the corresponding S-RBD_CoV‑2_/LY-CoV016 mAb complexes. Colors and other explanations as in Fig. 3. The numerical values of ΔΔG, all related energy terms, and all underlying intermolecular intramolecular interactions are reported in Tables S19–S30 and Figures S11–S22.

**Figure 7:**
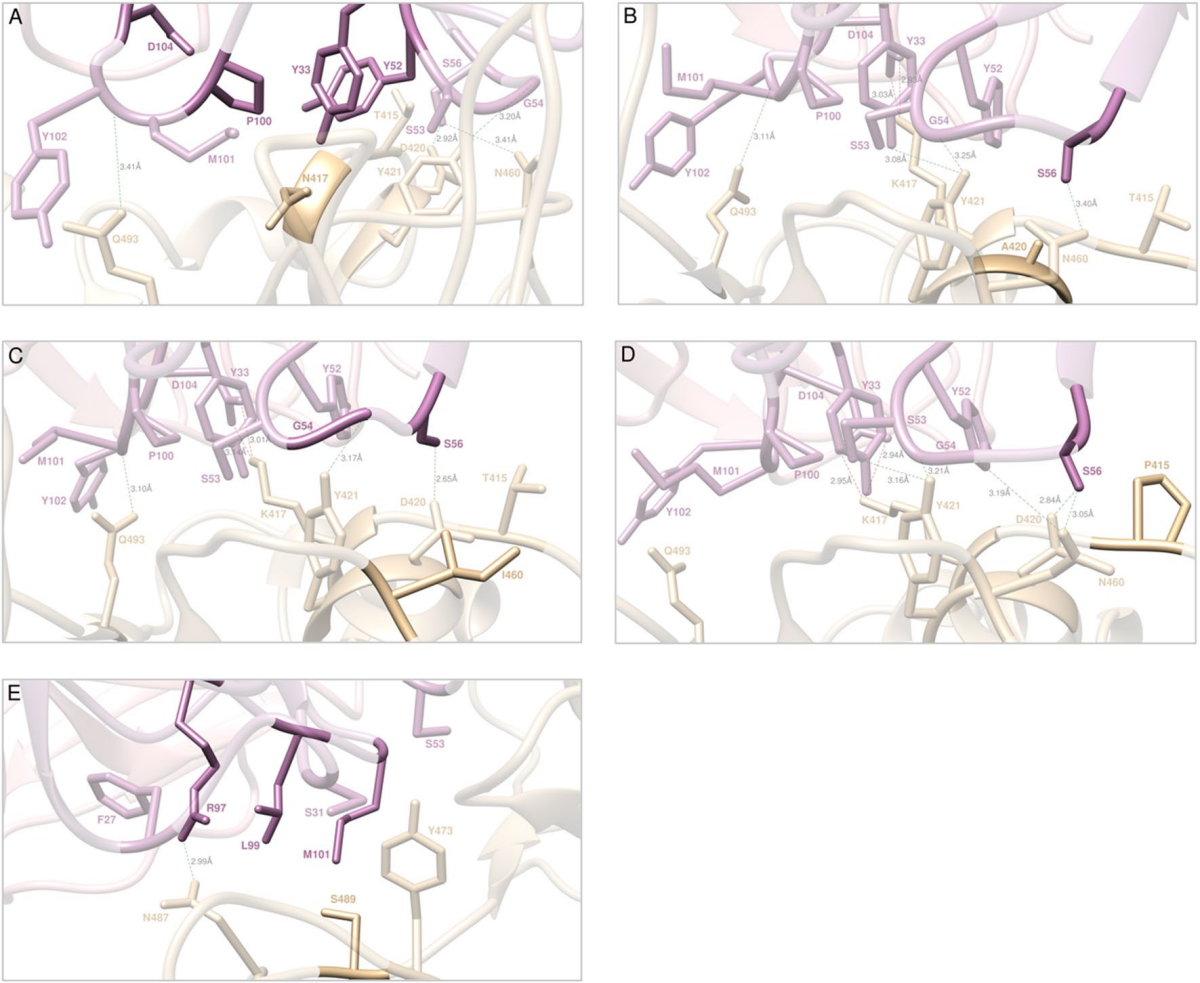
Main interactions involving the S-RBD_CoV‑2_ K417N (**A**), D420A (**B**), N460I (**C**), T415P (**D**), and Y489S (**E**) mutants at the interface with the LY-CoV016 (etesevimab) mAb as obtained from the relevant equilibrated MD simulations. Images for all other circulating mutants (K417E/R/T, D420G/, N460K/S/T, T415A/I/N/S and Y489C/F/H) are shown in Figures S11–S14 and S18 (see also Tables S19–S22 and S26 for details). Colors and other explanations as in Fig. 5.

Our CAS data highlight the wild-type K417 as a hot-spot residue in the interaction between the S-RBD_CoV‑2_ and the LY-CoV016 mAb (Fig. 5B,D, Tables S15, S16). As such, it is not surprising that replacing K417 on the viral protein with each of the alternative circulating mutants (K417E/N/R/T) reflects into a very strong interface disrupting behavior, with the exception of the substitution K417R, for which our *in silico *mutagenesis data anticipate a neutral effect (Figs. 6A, 7A and Fig. S11, Tables S18, S19). As seen in Fig. 7A for the K417N mutant as a paradigm, the current MD simulations show that both the double SB with the side chain of LY-CoV016 D_H_104 and the HB between the charged amine group of K417 and the hydroxyl moiety of Y_H_52 cannot longer be detected in the MD trajectory of the mutant complex. Also, the K417 network of underlying CIs involving a polar interaction with Y_H_33 and a hydrophobic contact with P_H_100 is likewise perturbed when K is replaced by N at the same position (Fig. 7A, Table S19). Quite importantly, in the same binding region the presence of N417 affects other protein/protein interactions, including the absence of the three stabilizing HBs between N460 and G_H_54, T415 and S_H_56, and Y421 and S_H_53, respectively (Fig. 7A, Table S19). These evidences ultimately translate into a drastically lower affinity of the N417 mutant spike protein for the LY-CoV016 mAb (ΔΔG_CoV-2_(K417N) = − 7.27 ± 0.07 kcal/mol, Fig. 6A and Table S18). The effects observed for the E417 and T417 spike mutants are completely similar to those just described for the N417 isoform, although in both these cases the interface HB between the side chains of N460 and G_H_54 is again detected yet at the expenses of the analogous interaction between the viral Q493 and the mAb Y_H_102, which is missing along the entire MD trajectories of the corresponding supramolecular assemblies. As such, the variation in binding free energy between the wild-type and a mutant spike protein carrying either E or T at position 417 in complex with the LY-CoV016 mAb is predicted to be quite significant (ΔΔG_CoV-2_(K417E) = − 7.56 ± 0.18 kcal/mol and ΔΔG_CoV-2_(K417T) = − 7.14 ± 0.09 kcal/mol, Fig. 6A, Table S18). On the other hand, only minor interface perturbations are observed in the presence of the R417 mutation (Figure S11, Table S19), in line with the predicted small change in protein/protein affinity (ΔΔG_CoV-2_(K417R) = − 0.99 ± 0.15 kcal/mol, Fig. 6A, Table S18).

#### D420 and N460

Converting the SARS-CoV-2 spike residues D420 and N460 in alanine via CAS analysis suggests that these two mutant isoforms induce only limited perturbing effects at the relative S-RBD_CoV‑2_/LY-CoV016 mAb binding interface (Fig. 5B,D, and Table S16). Surprisingly, however, the computational mutagenesis data for all circulating viral mutations at these two spike positions (D420A/G/N and N460I/K/S/T) reveal strong interface-destabilizing effects in all cases, with difference in free energy of binding with respect to the wild-type protein ranging from ~ − 5 to ~ − 3 kcal/mol (i.e., ΔΔG_CoV-2_(D420A) = − 4.36 ± 0.17 kcal/mol, ΔΔG_CoV-2_(D420G) = − 4.39 ± 0.10 kcal/mol, ΔΔG_CoV-2_(D420N) = − 4.23 ± 0.10 kcal/mol, ΔΔG_CoV-2_(N460I) = − 5.01 ± 0.14 kcal/mol, ΔΔG_CoV-2_(N460K) = − 3.28 ± 0.18 kcal/mol, ΔΔG_CoV-2_(N460S) = − 4.06 ± 0.09 kcal/mol, and ΔΔG_CoV-2_ N460T) = − 4.12 ± 0.14 kcal/mol) (see also Table S18). The molecular rationale for these results relies not only on the fact all D420 and N460 S-RBD_CoV‑2_ variants remove all direct interactions provided by aspartic acid (420) or glutamine (460) but also exert a domino effect on the nearby spike residues populating the same binding region, including T415 and, above all, the hot spot K417. Considering A420 as an exemplar of all D420 mutant behavior, from Fig. 7B it is quickly seen that the wild-type HB with the side chain of S_H_56 is evidently missing, as are the three topical HBs between T415 and S_H_56, between K417 and Y_H_52, and between N460 and G_H_54, respectively (see also Figure S12 and Table S20). Similarly, taking I460 as a proof-of-concept for the N460 variants, the relevant MD trajectory reveals the absence of the same HBs engaged by the side chains of T145 and K147 on the viral spike and those of S_H_56 and Y_H_52 on the Ab, respectively. At the same time the direct wild-type 460 intermolecular HBs with G_H_54 and S_H_56 are obviously suppressed in the I460 Spike mutant/mAb complex, along with the loss of the same interaction between Y421 and S_H_53 across the respective protein/protein interface (Fig. 7C and Fig. S13, Table S21).

#### T415 and Q493

Although these two SARS-CoV-2 S protein residues belong to the first binding region centered around two key viral amino acids in the stabilization of the S-RBD_CoV‑2_/LY-CoV016 mAb interface—N460 and K417, respectively (Fig. 5D)—the ΔΔG values currently predicted for replacement of both these spike positions with all reported variants (T415A/I/N/S and Q493H/K/L/R) indicate only moderate interface perturbation outcomes, with the notable deviation of the T415P mutant, for which a robust loss in affinity of this viral variant for the mAb is anticipated (Fig. 6D,E and Fig. S14, S15, Tables S18 and S22, S23). In detail, while the conservative mutation T415S ensues the preservation of the wild-type interaction network, in the case of the T415A/I/N variants the analysis of the present simulations shows that the two spike-mAb anchoring intermolecular HBs in which the wild-type residue is involved (i.e., T415-S_H_56 and K417-Y_H_52, Fig. 5D) cannot longer be detected in the trajectory of the mutant complexes. However, the extensive underlying network of other SBs, HBs, and CIs remains almost unaffected across the corresponding binding interfaces (Figure S14, Table S22), ultimately resulting in a limited decrement of the corresponding free energy variations (Fig. 6D, Table S18). In the case of the T415P variant, the remarkably negative effect on spike/mAb affinity predicted by our in silico mutagenesis is sensibly linked—aside for the same perturbating effects just discussed for the other mutations at the same viral protein location—to the absence of the additional interface HB and CIs between the side chains of Q493 on the spike and of Y_H_102 on the mAb HC (Fig. 7D, Table S22). Accordingly, the S-RBD_CoV‑2_ T415P mutation reported so far in circulating viral populations is predicted to be potentially destabilizing for the S-RBD_CoV‑2_/LY-CoV016 interface (ΔΔG_CoV-2_(T415P) = − 2.83 ± 0.07 kcal/mol, Fig. 6D and Table S18).

#### Y473, N487 and Y489

These viral residues belong to the spike/LY-Cov016 binding region that, according to the relevant MD trajectories, is characterized by an important network of stabilizing hydrogen bonds. Nevertheless, the present computational mutagenesis data report only neutral-to-mild interface destabilizing effects for the circulating SARS-CoV-2 RBD variants of Y473 (Y473F/H) and N487 (N487D) (see Table S18, Figures S16, S17, and Tables S24, S25 for details). Briefly, in the case of the Y473F mutation the loss of the HB between the wild-type tyrosine and the side chain of S_H_31 on the LY-CoV016 mAb HC (Fig. 5E) detected in the MD trajectories of all variants has only minor effects on all other important intermolecular interactions populating same region, while the phenylalanine-to-histidine mutation is virtually conservative (ΔΔG_CoV-2_(Y473F) = − 1.67 ± 0.08 kcal/mol and ΔΔG_CoV-2_(Y473H) = − 0.19 ± 0.16 kcal/mol, respectively, Table S18, Figure S16, Table S24). The predicted minor loss in binding affinity of the D487 spike variant for the LY-CoV016 mAb (ΔΔG_CoV-2_(N487D) = − 0.70 ± 0.09 kcal/mol, Table S18 on the other hand, is the result of a compensatory effect as the mutant aspartic acid provides a permanent intermolecular SB with the guanidine group of the mAb R_H_97 that makes up for the loss of the two HBs between Y473 and S_H_31 and Y489 and R_H_97, respectively (Fig. 5E and Fig. S17, Table S25). Finally, according to our MD analysis the circulating Y489 S-RBD_CoV‑2_ variants induce a moderate decrease in affinity of the viral spike protein for the LY-CoV016 mAb (Fig. 6F, Table S18). In particular, the conversion of tyrosine 489 into cysteine or serine results in the abrogation of the direct HB with R_H_97 as well as the hydrophobic contact with L_H_99. Moreover, the HB involving Y473 and S_H_31 is also missing along the entire MD trajectories of the Y489C and Y489S S-RBD_CoV‑2_ mutant proteins, as shown in Fig. 7E for the S489 isoform (see also Figure S18 and Table S26). In line with this, the calculated ΔΔG values numerically support moderate interface destabilizing effects upon substitution of the wild-type tyrosine with these two residues (ΔΔG_CoV-2_(Y489C) = − 2.15 ± 0.10 kcal/mol, and ΔΔG_CoV-2_(Y489S) = − 2.41 ± 0.07 kcal/mol, respectively, Fig. 6F and Table S18).

#### E406, L455, F456 and Y505

The actual computational data for mutating these four viral protein residues into the SARS-CoV-2 circulating variants (E406D/Q, L455F/S/V, F456L/Y and Y505F/H/W) account for neutral-to-mildly negative effects on the stability of the corresponding S-RBD_CoV‑2_/LY-CoV016 mAb binding interface, with estimated ΔΔG values all below 1 kcal/mol for all alternative amino acids considered (Fig. 6G–J, see also Table S18, Figures S19–S22 and Tables S27–S30). Therefore, all these SARS-CoV-2 spike position variants do not appear to have a significant role in escaping the LY-CoV016 antibody.

## Discussion

The purpose of this work was to provide an *in silico* molecular rationale of the role eventually played by currently circulating S-RBD_CoV‑2_ mutations in evading the immune surveillance effects elicited by the two Eli Lilly LY-CoV555/bamlanivimab and LY-CoV016/etesevimab monoclonal antibodies. Table 2 summarizes the main findings from this study and shows that, compared to the wild-type SARS-CoV-2 spike protein, all mutants highlighted in light or dark red are predicted to be markedly more resistant to neutralization by both these mAbs, those shown in yellow might exert only mildly perturbing protein/protein binding, while those listed in gray are not likely to confer any mAb escaping advantage to the viral protein.

**Table 2:**
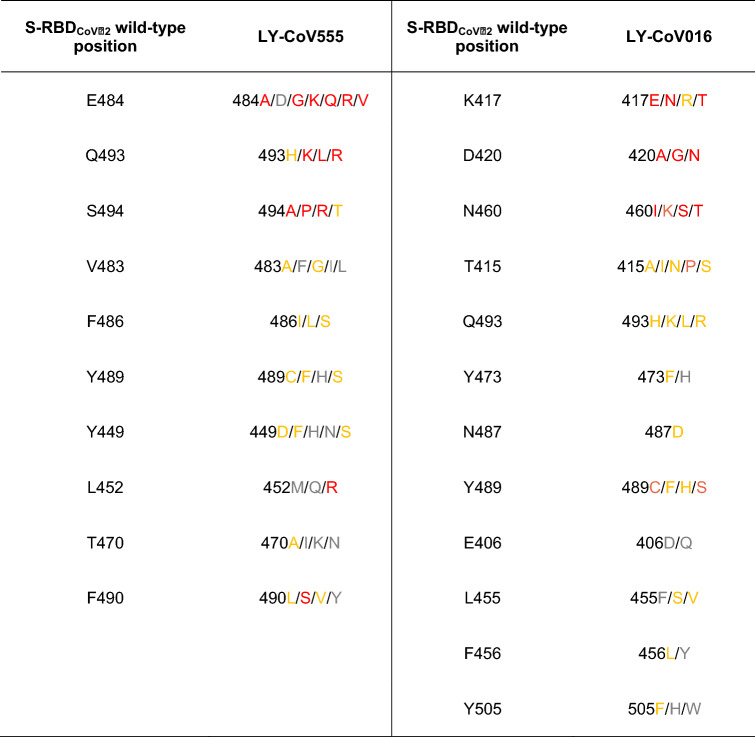
Color-code ranking of circulating SARS-CoV-2 spike protein mutants with respect to their predicted resistance to neutralization by LY-CoV-555 (bamlanivimab) and LY-CoV016 (etesevimab) monoclonal antibodies. Colors as in Table 1.

According to the” Fact sheet for health care providers—emergency use authorization (EUA) of bamlanivimab and etesevimab”^58^, resistant variants to both mAbs were already reported by Eli-Lilly researchers on March 18, 2021 using S-protein directed evolution and serial passages in cell cultures of SARS-CoV-2 in the presence of either antibody. On the other hand, resistant variants were not reported when the two mAbs were tested together using the same methodology. Spike variants identified in these studies that presented reduced susceptibility to the LY-CoV555 mAb included the following substitutions: E484D/K/Q, F490S, Q493R, and S494P. Concerning the spike position 484, after our CAS approach identified E484 as a key player residue at the S-RBD_CoV‑2_/LY-CoV555 binding interface (Fig. 2A,B, Table S2), we considered all possible mutations actually reported at this position in circulating viral variants (i.e., E484A/D/G/K/Q/R/V), and found that all these amino acid variations should confer strong escaping ability to bamlanivimab (Figs. 3A, 4A and Fig. S1, Table 2 and Tables S4, S5). From a validation standpoint, the E484K mutation is present in a large number of VOC/VOI/VUM—including the lineages B.1.525 (now Eta, firstly reported in Nigeria on 12/20), P.1 (now Gamma) and P.2 (now Zeta, Brazil, 12/20), P.3 (now Theta, The Philippines, 01/21), B.1.351 (now Beta, South Africa, 09/20), B.1.621 (Colombia, 01/2021), and some strains of lineages B.1.1.7 (firstly reported in the United Kingdom on 09/20, now Alpha) and B.1.526 (reported on 11/20 in the city of New York, USA, now Iota)^51,52^—and it is indeed well known to confer substantial loss of sensitivity to neutralizing Abs found in sera of convalescent and vaccinated individuals^59–70^. Further, for all these variants there is evidence of a significant reduction in neutralization by the LY-CoV555/LY-CoV016 and other mAb treatments^56,70–74^. Collier and coworkers very recently reported that the introduction of the E484K mutation in the B.1.1.7 background (to account for the new VOC B.1.1.7 + E484K found in the virus isolated both in UK and in Pennsylvania, USA)^75^ led to robust loss of neutralizing activity by 19 out of 31 vaccine-elicited antibodies and mAbs if compared with the decrease in sensitivity conferred by the mutations in B.1.1.7 alone^76^. Moreover, the E484Q/V/A/G/D mutations have been just described by Chen et al. as critical in promoting escape not only from Eli Lilly mAbs but also from other similar therapeutics that are currently in clinical trials^77^.

Mutating the wild-type spike F490 into alanine also flagged this position as a residue affording an important contribution to the protein/protein interface (Fig. 2A,B, Table S2). Interestingly, the corresponding mutagenesis into all reported variants (F490L/S/V/Y) revealed that only the F490S spike mutant is a potential escapee for LY-CoV555 (Fig. 3J and Fig. S10, Tables S4 and S14), in agreement with Lilly’s and other experimental observations^58,64,77^. Of note F490S, although listed in the actual spike circulating mutations, is not a component of any VOC or VOI listed so far^51,52^. Finally, CAS predicted viral spike residues Q493 and S494 to be the two remaining hot spots at the viral protein/bamlanivimab binding interface (Fig. 2A,E, Table S2). In silico mutagenesis of Q493 and S494 into the circulating variants (Q493H/K/L/R, and S494A/P/R/T) not only confirms Lilly’s data about Q493R and S494P as resistant mutations for LY-CoV555^58^ but also predicts a potential role of other substitutions at these two S-protein positions (i.e., Q493K/L and S494A/P/R) in mediating evasion to this mAb (Figs. 3B,C, 4B,C, and Fig. S2, S3, Table 2 and Tables S6, S7). In line with these predictions, three new studies highlighted all these mutants as viral proteins that may hinder the efficiency of existing vaccines and expand in response to the increasing after-infection or vaccine-induced seroprevalence^64,77,78^. Remarkably, the spike S494P mutation is a component of the B.1.17 + S494P VOC^51^/VUM^53^ identified in United Kingdom in January 2021.

In the fact sheet produced by Lilly^58^ the spike 452 position was not mentioned as a possible site of LY-CoV555 escaping mutant per se. However, L452R is a spike mutation of interest (MOI)^52^ present in the VOC lineages B.1.427/B.1.429 (reported in California, USA, on 09/20, now Epsilon), B.1.526.1 (New York City, USA, 10/20, Iota subtype), and in the B1.617.1 (Kappa)/B.1.617.2 (Delta)/B.1.617.3 lineages now rapidly and deadly spreading in India (12/20–02/21), where it is always found along with the D614G substitution. Importantly, the L452R mutation is also present in tandem with E484Q, in particular in the B.1.617.1 (Kappa) variant that is responsible for actual disease outbreaks in 49 countries in all six WHO regions^4^. Using a pseudo-virus expressing the spike protein from the B.1.427/B.1.429 (Epsilon) lineages, or the L452R substitution only, however, the researchers at Lilly reported reduced susceptibility to bamlanivimab and etesevimab together of 7.7-fold or 7.4-fold, respectively^58^. Further experimental works^79–82^ already reported increased viral load/transmissibility and escape ability from neutralizing antibodies for this variant when tested against vaccine-elicited sera. Actually, in their preprint work Hoffmann et al. analyzed whether the SARS-CoV-2 VOC B.1.617 is more adept in entering cells and/or evade Ab responses^83^. They found that B.1617 entered two out of 8 cell lines tested (specifically, the human lung- and intestine-derived Calu-3 and Caco-2 cell lines, respectively) with slightly increased efficiency, and was blocked by an entry inhibitor. However, in stark contrast, B.1.617 was found to be fully resistant to LY-CoV555 and partially resistant against neutralization by Abs elicited upon infection or vaccination with the Comirnaty/Pfizer-BioNTech vaccine. Our present data support the escaping potential of the L452R viral mutation with respect to bamlanivimab (Fig. 3H, 4D, and Fig. S8, Table 2 and Table S12), while we did not detect any effect in terms of changed affinity of this mutant protein toward etesevimab. Moreover, our data also suggest that the co-presence of the E484Q (Fig. 3A and Fig. S1, Table 2 and Table S5) may synergistically contribute in rendering the two B.1.617.1 and B.1.617.3 variants potent evaders of antibody surveillance.

Concerning the alternative LY-CoV016 mAb, the official Lilly’s fact sheet^58^ reports that SARS-CoV-2 spike mutants showing reduced susceptibility to etesevimab include substitutions K417N, D420N, and N460K/S/T. In agreement with this and other evidences^71–78^, our current computational alanine/mutagenesis study marks K417 and all its reported variants (K417E/N/R/T) as the strongest hot spots in eliciting potential escape to the LY-CoV016 mAb (Figs. 5B, 6A, 7A, and Fig. S11, Table 2 and Table S19). Of note, the K417N and K417T in particular are spike MOIs in the SARS-CoV2 VOC lineages B.1.351 (Beta) and P.1 (Gamma), respectively. Similarly, not only the D420N but all reported circulating spike mutations at positions 420 are predicted by our study to be endowed with high LY-CoV016 escaping potential (Fig. 6B, 7B, and Fig. S12, Table 2 and Table S20), in line with recent findings^78^. Finally, and in full agreement with Lilly’s data, LY-CoV016 is also found to be escaped by all spike N460 variations (N460I/K/S/T) (Figs. 6C, 7C and Fig. S13, Table 2 and Table S21). In addition, in the current study we identify three further single amino acid changes along the primary sequence of SARS-CoV-2 spike protein that—although not reported as current VOC/VOI/VUM—could escape the action of LY-CoV016, that is the T415P and the Y489C/S mutations (Figs. 6D,F, 7D,E, Figs. S14 and S18, Table 2, Tables S22 and S26). Since these spike mutants are present in circulating viral variants, in our opinion they should be taken into consideration as they might limit the therapeutic usefulness of this mAb, both per se and in its cocktail combination with LY-CoV555.

As a conclusive remark concerning the available anti-SARS-CoV-2 vaccines, according to the report by Andreano and Rappuoli published on May 10, 2021 in Nature Medicine^84^ the efficacy of the FDA/EMA approved Ad26.COV2-S vaccine (now Janssen COVID-19 Vaccine) and the EMA approved Oxford–AstraZeneca ChAdOx1 (now Vaxzevria) against the variant B.1.351 (South Africa now Beta, with E484K, K417N and N501Y as spike MOIs) decreased from 85 to 57% and from 62 to 10%, respectively. In parallel, the titer neutralizing antibodies induced by the m-RNA vaccines approved by both governmental agencies (i.e., the BNT162b2 Pfizer/BioNTech COVID-19 vaccine/Comirnaty and COVID-19 vaccine Moderna) against the same SARS-CoV-2 variant is reported to decline by 7- to 12 -fold, while no negative effect on neutralization is seen for the B.1.1.7 (Alpha) variant (with N501Y/D614G as spike MOIs). Additionally, the work of Planas and collaborators documented low titers of neutralizing antibodies against the B.1.351 (Beta) variant in a cohort of 19 individuals after both doses of the Comirnaty vaccine^85^. In all these cases, the spike E484K mutation appears to be the real key player in reducing neutralization by antibodies induced by the vaccines. And this, in turn, support the view that vaccination elicits a natural infection-like antibody response, and that spike variants like E484K may spread as antigenic evolutions of SARS-CoV-2 to efficiently evade this response. On the bright side, all vaccines currently approved appear at least to protect from the severe forms of infection^86,87^, and second-generation vaccines and mAbs aiming at containing VOC spreading are under investigation^88^.

In concluding this work, we report that a challenge of our global *in silico* results against the relevant experimental data just published by the Starr group^56^ resulted in an overall 90% agreement. Starr and coworkers used a deep mutational scanning method to map variants in the SARS-CoV-2 RBD that elude antibody binding. In a nutshell, this method entails displaying almost all SARS-CoV-2 RBD residue mutants on the yeast surface, incubating the yeast with the mAb, exploiting fluorescence-activated cell sorting (FACS) to enrich functional, mAb-escaping SARS-CoV-2 RBD mutants, and using deep sequencing to quantify the extent to which each mutation is enriched in the mAb-evading population with respect the original sample. Then, again according to the work of Starr et al., the impact of each SARS-CoV-2 RBD variant on the relevant interaction with the mAb is quantified by calculating its "escape fraction" X_esc_, i.e., the fraction of yeast expressing the mutant that falls into the antibody-escape FACS bin. Accordingly, X_esc_ varies from 0—for those SARS-CoV-2 RBD mutations having no effect on the mAb binding—to 1 for those viral spike RBD variants endowed with the strongest mAb-escaping ability^56^. In order to compare our predicted data on all naturally occurring and clinically significant spike RBD mutations with the corresponding X_esc_ values experimentally reported in^56^, we independently and *a* *priori* established and adopted the color-coded criterion based on the predicted ΔΔG range of values reported in Table 1. Then, before starting our entire computational campaign, the mutation ranking thus defined (Table 1) was again independently mapped onto the experimental X_esc_ values as follows: in silico neutral/mildly destabilizing mutations (− 2.00 ≤ ΔΔG ≤ + 0.25 kcal/mol) ⇒ 0 ≤ X_esc_ ≤ 0.5, and in silico destabilizing/highly destabilizing mutations (− 4.00 ≤ ΔΔG < − 2.00 kcal/mol) ⇒ 0.5 < X_esc_ ≤ 1.0. Finally, by comparing each estimated ΔΔG data with the corresponding X_esc_ values we attained a 90% of agreement between in silico predicted and experimentally determined ability of each clinically relevant circulating S-RBD_CoV‑2_ variant to escape both LY-CoV555 and LY-CoV016 neutralization.

Thus, the results presented here provide a molecular-based rationale for all relative experimental findings, constitute a fast and reliable tool for identifying and prioritizing all present and newly reported circulating spike SARS-CoV-2 variants with respect to antibody neutralization, and yield substantial structural information for the development of next-generation vaccines and mAbs more resilient to viral evolution. In addition, this achievement leads us to conclude that the current circulating SARS-CoV-2 and all possible emergent variants carrying these mutations in the spike protein can present new challenges for mAb-based therapies and ultimately threaten the fully-protective efficacy of currently available vaccines. Importantly, the computational procedure described in this paper has a truly general character, and can be applied to reliably predict the effect of mutations on other protein/protein (as well as protein/ligand and protein/nucleic acid) interactions playing key roles in the pathogenesis of different, major human diseases including, e.g., bacterial infections, hereditary syndromes and, above all, cancer, as previously shown by our research group^89–96^.

## Methods

### Computational methods

The starting structure for the wild type SARS-CoV-2 S-protein receptor binding domain (S-_RBDCoV-2_) in complex with either LY-CoV555 or LY-CoV016 were obtained from the RCSB Protein Data Bank^97^ (PDB ID 7KMG^38^ and 7C01^39^, respectively). The physiological protonation state for all residues in each system was obtained by the H++ server^98^ (http://biophysics.cs.vt.edu/H++).

The *tleap* software provided within AMBER20^99^ was used for the parametrization of each system, assigning the ff14SB^100^ and GLYCAM06j-1^101^ forcefields to the protein and glycan structures. The complexes were next solvated in a box of TIP3PB^102^ water molecules spanning at least 1.5 nm from each solute atom. An appropriate number of Na^+^ and Cl^−^ atoms were added to neutralize the system and mimic the physiological salt concentration of 0.15 M. The following simulation scheme was applied for each simulated system. While applying a weak restraint (10 kcal/mol) on the proteins’ backbone atoms, the simulation box was firstly energy minimized (this and the following minimization steps were composed of 3000 steps of steepest descent followed by 3000 steps of conjugated gradient algorithms), than heated to 150 K in 10 ps of canonical ensemble (NVT) molecular dynamics (MD), followed by another 50 ps MD simulation in the isothermal/isobaric ensemble (NPT, P = 1 atm, maintained by the Berendsen barostat^103^) to reach the target temperature of 300 K. The restraints were then gradually removed in 5 steps (− 2 kcal/mol per step) of energy minimization. The MD simulation was next carried out without restraints for further 10 ns in NPT conditions (*phase 1*); after this time interval, the MD data production run was further continued up to 1 μs, during which pressure was maintained using the Monte Carlo barostat implemented in AMBER (*phase 2*). Along the entire MD trajectory, electrostatic interactions were computed by means of the particle mesh Ewald (PME) algorithm^104^, temperature was regulated by the Langevin method^105^ (collision frequency of 3 ps-1). The SHAKE algorithm ^106^ was applied to allow a 2 fs integration time step. All calculations were run with the *pmemd* module of AMBER20 running on the supercomputer Marconi100 (CINECA, Bologna, Italy) and on our CPU/GPU hybrid cluster. All images were produced by the UCSF Chimera software^107^, VMD software^108^ and on Prism 8 GraphPad Prism version 8.0.0 for Mac (GraphPad Software, San Diego, California USA, http://www.graphpad.com).

After the first 5 ns of the *phase 2* MD trajectory, 5 ns MD data were selected to calculate enthalpy and entropy contributions. Configurational sampling in this part of the simulation was preformed accordingly, with a time step of 10 fs; thus, a total of 500,000 snapshots, sufficient for the interaction entropy (IE) calculations^55,57,109–111^, were extracted from the relevant MD trajectory for the calculation of the protein/protein residue- specific interactions. The free energy was calculated for each molecular species in the framework of the MM/PBSA ansatz^112^, and the protein/antibody binding free energy was computed as the difference:

1 $$\Delta G = G_{{S - RBD_{COV2} /mAb}} - \left( {G_{{S - RBD_{COV2} }} + G_{mAb} } \right) = \Delta E_{vdW} + \Delta E_{ELE} + \Delta G_{SOL} - T\Delta S = \Delta H - T\Delta S.$$

Here $\Delta E_{vdW}$ and $\Delta E_{ELE}$ represent van der Waals and electrostatic molecular mechanics energies, and $\Delta G_{SOL}$ includes the solvation free energy. The internal dielectric constant was set to the values of 2, 3 and 9 for nonpolar, polar, and charged residues, respectively^109,110,113,114^. Lastly, the entropic contribution ($T{\Delta }S$) was explicitly computed from the MD simulation by using the Interaction Entropy (IE) method^55,57,109–111^.

The role of the protein/protein interface key residues was studied by performing computational alanine scanning (CAS) experiments^55,113,114^. Accordingly, the absolute binding free energy of each mutant receptor—in which each key residue was replaced by alanine by truncating the mutated residue at the $C_{\gamma }$ atom, and replacing it with a hydrogen—was calculated with the MM/PBSA method. Accordingly, the difference in the binding free energy between the wild-type (WT) protein and its alanine mutant (ALA) counterpart, $\Delta \Delta G_{CAS}$, is given by:

2 $$\Delta \Delta G_{CAS} = \Delta G_{WT} - \Delta G_{ALA} .$$

Thus, the CAS methodology allows for the estimation of the contribution of a given residue with respect to the overall protein–protein binding free energy; indeed, according to the equation, a negative value of $\Delta \Delta G_{CAS}$ indicated a favorable contribution for the wild type residue in that position and vice versa.

The role of the protein/mAb interface key residues mutations was calculated with the MM/PBSA method^57^. Accordingly, the difference in the binding free energy between the wild-type (WT) protein and mutant counterpart, $\Delta \Delta G$, is given by:

3 $$\Delta \Delta G = \Delta G_{WT} - \Delta G_{MUTANT} .$$

Thus, the adopted methodology allows for the estimation of the contribution of a given residue with respect to the overall protein/mAb binding free energy; indeed, according to the equation, a negative value of ${{\Delta \Delta }}G$ indicated a favorable contribution for the wild type residue in that position and vice versa.

At the structural level, the stability of the main protein/protein interface intermolecular and intramolecular interactions detected during the MD simulation time interval adopted for the energetic analysis was assessed along the entire duration of the MD run.

## Supplementary Information

Supplementary Information.
