# Supplementary material for: Molecular rationale for SARS-CoV-2 spike circulating mutations able to escape bamlanivimab and etesevimab monoclonal antibodies
Source: Sci Rep. 2021 Oct 12;11:20274. doi: 10.1038/s41598-021-99827-3 (PMC8511038; doi:10.1038/s41598-021-99827-3)
Supplement: Supplementary file 1 — Supplementary Information. [file 41598_2021_99827_MOESM1_ESM.pdf]

Supplementary Information for

Molecular Rationale for SARS-CoV-2 Spike Circulating Mutations Able to Escape Bamlanivimab and Etesevimab Monoclonal Antibodies

Erik Laurini[1,‡], Domenico Marson[1,‡], Suzana Aulic[1], Alice Fermeglia[1], Sabrina Pricl[1,2,*]

*Sabrina Pricl
Email: sabrina.pricl@dia.units.it

This PDF file includes:

Figures S1 to S22
Tables S1 to S30

Table S1. Main intermolecular and intramolecular interactions between residues at the protein-protein interface detected during MD simulations of the LY-CoV555 antibody in complex with the RBDs of SARS-CoV-2 S-RBD (COV2). HB = hydrogen bond; SB = salt bridge; CI = contact interactions, including van der Waals/hydrophobic (vdW/h), polar (p), π/π and π/cation (π/c) interactions. In the HB column, s-s indicates side chain-side chain interactions while s-b or b-s indicate side chain-backbone or backbone- side chain interactions, respectively.

HB	COV2	Length (Å)	LY-CoV555
s-s	Q493	3.31 ± 0.12	R_H104
s-b		3.34 ± 0.17	R_H104
s-s		3.25 ± 0.18	E_H102
s-s	S494	3.18 ± 0.19	R_H104
s-s		2.86 ± 0.16	E_H102
**SB**	**COV2**	**Length (Å)**	**LY-CoV555**
	E484	2.74 ± 0.10	R_H50
		3.05 ± 0.14	
	E484	2.82 ± 0.11	R_L96
		2.96 ± 0.13	
**SB**	**LY-CoV555**	**Length (Å)**	**LY-CoV555**
s-s	E_H102	2.76 ± 0.11	R_H104
**CI**	**COV2**	**LY-CoV555**	
p	Y449	T_H28	
p		S_H30	
p		N_H31	
vdW/h		I_H54	
vdW/h	L452	I_H54	
vdW/h		L_H55	
vdW/h	T470	L_H55	
vdW/h		I_H57	
vdW/h	V483	W_H47	
vdW/h		R_H50	
vdW/h		N_H59	
vdW/h		T_L94	
vdW/h		R_L96	
vdW/h	E484	Y_H101	
vdW/h		Y_H110	

π/π	F486	Y_L32
π/π		Y_L92
vdW/h	Y489	Y_H110
p		Y’32
vdW/h	F490	I_H52
vdW/h		L_H55
vdW/h		I_H57
π/π		Y_H101
p	S494	N_H31

Table S2. Relative binding free energy and its components calculated by the combined computational alanine scanning mutagenesis – interaction entropy approach for the S-RBD of SARS-CoV-2(COV2) residues effectively involved in the binding interface with the LY-CoV55 antibody (see the SI Materials and Methods section for details). IE = interaction entropy. $\Delta\Delta G = \Delta G_{WILDTYPE} - \Delta G_{ALA}$ (see text for details).

	**Y449A**	**L452A**	**T470A**	**V483A**	**E484A**	**F486A**	**Y489A**	**F490A**	**Q493A**	**S494A**
$\Delta\Delta E_{DISP}$	-0.50	-0.76	-0.42	-1.67	-0.63	-0.76	-0.54	-2.49	-0.46	-0.21
$\Delta\Delta E_{ELE}$	-1.22	0.09	-0.19	0.09	-5.57	-0.77	-0.49	-0.07	-3.93	-3.66
$\Delta\Delta H$	-1.72	-0.67	-0.61	-1.58	-6.20	-1.53	-1.03	-2.56	-4.39	-3.87
$\Delta\Delta IE$	-0.21	-0.09	-0.03	-0.12	0.28	0.09	-0.09	0.18	0.21	-0.15
**$\Delta\Delta G_{cov2}$**	**-1.93 (0.16)**	**-0.76 (0.11)**	**-0.64 (0.15)**	**-1.70 (0.18)**	**-5.92 (0.12)**	**-1.44 (0.15)**	**-1.12 (0.09)**	**-2.38 (0.22)**	**-4.18 (0.14)**	**-4.02 (0.12)**

Table S3. Relative binding free energy and its components calculated by the combined computational alanine scanning mutagenesis – interaction entropy approach for LY-CoV555 antibody residues effectively involved in the binding interface with the S-RBD of SARS-CoV-2 (COV2) (see the SI Materials and Methods section for details). IE = interaction entropy. $\Delta\Delta G = \Delta G_{WILDTYPE} - \Delta G_{ALA}$ (see text for details).

	**N_H31A**	**R_H50A**	**L_H55A**	**Y_H101A**	**E_H102A**	**R_H104A**	**Y_H110A**	**Y_L32A**	**Y_L92A**	**R_L96A**
$\Delta\Delta E_{DISP}$	0.12	-0.22	-1.15	-0.56	-1.35	-1.27	-0.79	-0.24	-0.32	-0.48
$\Delta\Delta E_{ELE}$	-1.57	-3.01	0.07	-0.89	-3.19	-3.56	0.06	-1.14	-0.46	-2.29
$\Delta\Delta H$	-1.45	-3.23	-1.08	-1.45	-4.54	-4.83	-0.73	-1.38	-0.78	-2.77
$\Delta\Delta IE$	-0.11	0.21	-0.02	0.11	0.22	0.25	0.12	-0.16	0.05	0.18
**$\Delta\Delta G_{cov2}$**	**-1.56 (0.14)**	**-3.02 (0.19)**	**-1.10 (0.08)**	**-1.34 (0.10)**	**-4.32 (0.13)**	**-4.58 (0.15)**	**-0.61 (0.09)**	**-1.54 (0.12)**	**-0.73 (0.17)**	**-2.59 (0.11)**

Table S4. Relative binding free energy and its components for the mutated S-RBD of SARS-CoV-2 (COV2) residues in the binding interface with the LY-CoV555 antibody. IE = interaction entropy. IE = interaction entropy. $\Delta\Delta G = \Delta G_{WILDTYPE} - \Delta G_{MUTANT}$ (see text for details).

	**Y449D**	**Y449F**	**Y449H**	**Y449N**	**Y449S**	**L452M**	**L452Q**	**L452R**	**T470A**	**T470I**	**T470K**	**T470N**
$\Delta\Delta E_{DISP}$	-0.52	-0.34	-0.22	-0.19	-0.75	-0.17	-0.10	-1.13	-0.42	0.21	0.18	0.09
$\Delta\Delta E_{ELE}$	-0.54	-1.13	0.03	0.32	-0.31	0.04	0.15	-4.28	-0.27	-0.12	-0.35	-0.20
$\Delta\Delta H$	-1.06	-1.47	-0.19	0.13	-1.06	-0.13	0.05	-5.41	-0.69	0.09	-0.17	-0.11
$\Delta\Delta IE$	-0.11	-0.04	0.10	0.08	0.05	-0.09	-0.21	0.12	0.08	-0.05	0.11	-0.01
$\boldsymbol{\Delta\Delta G_{COV2}}$	**-1.17**	**-1.51**	**-0.09**	**0.21**	**-1.01**	**-0.22**	**-0.16**	**-5.29**	**-0.61**	**0.04**	**-0.06**	**-0.12**
	**(0.15)**	**(0.18)**	**(0.16)**	**(0.09)**	**(0.12)**	**(0.13)**	**(0.14)**	**(0.15)**	**(0.19)**	**(0.10)**	**(0.17)**	**(0.12)**

	**V483A**	**V483F**	**V483G**	**V483I**	**V483L**	**E484A**	**E484D**	**E484G**	**E484K**	**E484Q**	**E484R**	**E484V**
$\Delta\Delta E_{DISP}$	-1.62	0.07	-0.34	0.41	0.43	-2.20	-0.23	-2.49	-2.01	-0.14	-2.12	-1.96
$\Delta\Delta E_{ELE}$	0.21	-0.34	-1.37	-0.16	-0.12	-3.86	-0.20	-4.92	-5.75	-2.41	-5.99	-4.03
$\Delta\Delta H$	-1.41	-0.27	-1.71	0.25	0.31	-6.06	-0.43	-7.41	-7.76	-2.55	-8.11	-5.99
$\Delta\Delta IE$	0.05	0.04	-0.27	-0.13	-0.16	-0.16	-0.18	-0.17	-0.07	0.02	0.12	-0.03
$\boldsymbol{\Delta\Delta G_{COV2}}$	**-1.36**	**-0.23**	**-1.98**	**0.12**	**0.15**	**-6.18**	**-0.61**	**-7.58**	**-7.83**	**-2.53**	**-7.99**	**-6.02**
	**(0.16)**	**(0.11)**	**(0.12)**	**(0.07)**	**(0.14)**	**(0.10)**	**(0.13)**	**(0.18)**	**(0.11)**	**(0.16)**	**(0.12)**	**(0.14)**

	**F486I**	**F486L**	**F486S**	**Y489C**	**Y489F**	**Y489H**	**Y489S**	**F490L**	**F490S**	**F490V**	**F490Y**	**Q493H**
$\Delta\Delta E_{DISP}$	-0.52	-0.74	-1.55	-0.05	-0.08	-0.09	-0.24	-0.97	-1.52	-1.38	-0.20	-0.78
$\Delta\Delta E_{ELE}$	0.05	-0.10	0.30	-0.31	-0.54	-0.05	-0.34	0.06	-0.93	-0.17	0.28	-1.33
$\Delta\Delta H$	-0.47	-0.84	-1.25	-0.36	-0.62	-0.14	-0.58	-0.91	-2.45	-1.55	0.08	-2.11
$\Delta\Delta IE$	0.16	0.12	0.14	-0.10	0.04	-0.11	0.09	-0.18	0.02	0.03	-0.01	0.16
$\boldsymbol{\Delta\Delta G_{COV2}}$	**-0.67**	**-0.72**	**-1.11**	**-0.46**	**-0.53**	**-0.25**	**-0.49**	**-1.09**	**-2.43**	**-1.52**	**0.07**	**-1.95**
	**(0.10)**	**(0.14)**	**(0.15)**	**(0.09)**	**(0.13)**	**(0.16)**	**(0.11)**	**(0.18)**	**(0.17)**	**(0.07)**	**(0.09)**	**(0.11)**

	**Q493K**	**Q493L**	**Q493R**	**S494A**	**S494P**	**S494R**	**S494T**
$\Delta\Delta E_{DISP}$	-1.25	-0.78	-1.00	-1.52	-0.87	-0.91	-0.26
$\Delta\Delta E_{ELE}$	-3.49	-3.61	-3.38	-4.17	-3.06	-5.09	-0.45
$\Delta\Delta H$	-4.74	-4.39	-4.38	-5.69	-3.93	-6.00	-0.71
$\Delta\Delta IE$	-0.09	0.13	-0.19	0.10	-0.23	0.19	0.01
$\boldsymbol{\Delta\Delta G_{COV2}}$	**-4.83**	**-4.26**	**-4.57**	**-5.59**	**-4.16**	**-5.81**	**-0.70**
	**(0.12)**	**(0.18)**	**(0.11)**	**(0.08)**	**(0.16)**	**(0.17)**	**(0.15)**

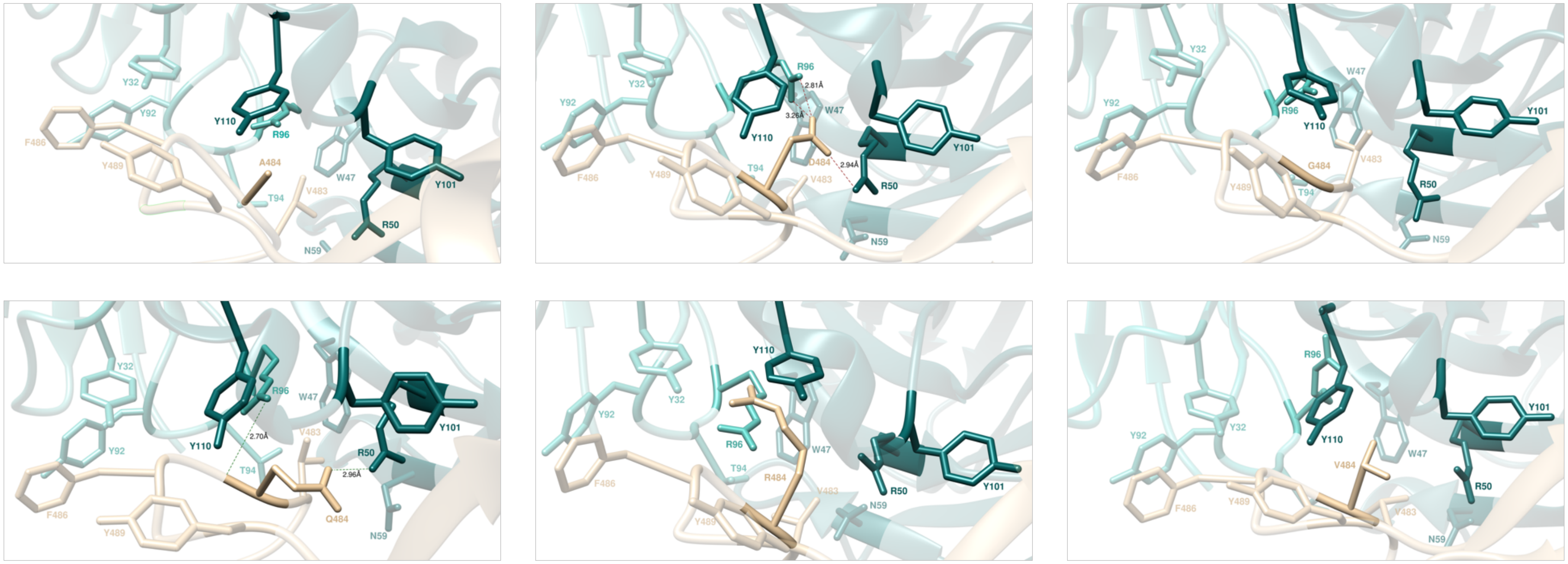

Fig. S1. Main interactions involving the S-RBD$_{CoV-2}$ A484 (top left), D484 (top middle), G484 (top right), Q484 (bottom left), R484 (bottom middle), and V484 (bottom right) at the interface with the LY-CoV555 antibody as obtained from the corresponding equilibrated MD simulations. The wild-type E484 and the K484 mutant complexes are presented and discussed in the main text (Figures 2D and 4A). In this and all remaining Figures, the secondary structure of the S-RBD$_{CoV-2}$ is shown as a light tan ribbon, while the heavy and light chains of the LY-CoV555 antibody are portrayed as light teal and light Tiffany green ribbons, respectively. Each protein residue under discussion and all other residues directly interacting with it are highlighted in dark matching-colored sticks and labelled; further residues/interactions related to the residue under investigation are evidenced in light matching-colored sticks and labelled in light gray. Hydrogen bonds and salt bridges directly involving the residue under discussion are represented as dark green and dark red broken lines, respectively, and the relevant average distances are reported (in black) accordingly. New HBs and SBs eventually detected in each mutant complex are also indicated using dark green/red broken lines and black labels. Further important HBs and SBs detected in each complex are also indicated using light green/red broken lines and light gray labels. For further details see Tables S1 and S5.

Table S5. Main interactions between the wild-type S-RBD$_{CoV-2}$ residue E484 and all considered mutants* at the protein-protein interface detected during MD simulations of the RBD of SARS-CoV-2 (COV2) in complex with the LY-CoV555 antibody. HB = hydrogen bond; SB = salt bridge; CI = contact interactions, including van der Waals/hydrophobic (vdW/h), polar (p), π/π and π/cation (π/c) interactions. In the HB column, s-s indicates side chain-side chain interactions while s-b or b-s indicate side chain-backbone or backbone-side chain interactions, respectively. Preserved/new or lost interactions are marked with the symbols ✓ and ✗,

respectively. Relevant changes in the type/nature of the interactions are indicated in parenthesis. For HBs and SBs, the relevant average lengths (in Å) are also reported (their standard deviations, all within 10%, are not shown for clarity). *Mutant K484 is discussed in detail in main text.

SB	COV2	LY-CoV555	E484	A484	D484	G484	K484	Q484	R484	V484
s-s	X484	R_H50	✓(2.74,3.05)	✗	✓(2.94)	✗	✗	✓(HB,2.96)	✗	✗
s-s	X484	R_L96	✓(2.82,2.96)	✗	✓(2.81,3.26)	✗	✗	✓(HB,s-b,2.70)	✗	✗
**CI**	**COV2**	**LY-CoV555**	**E484**	**A484**	**D484**	**G484**	**K484**	**Q484**	**R484**	
vdW/h	X484	Y_H101	✓	✓	✓	✗	✓	✓	✗	✓
vdW/h	X484	Y_H110	✓	✗	✓	✗	✓(p)	✓	✓(π/c)	✓
**CI**	**COV2**	**LY-CoV555**	**E484**	**A484**	**D484**	**G484**	**K484**	**Q484**	**R484**	**V484**
vdW/h	V483	W_H47	✓	✓	✓	✓	✓	✓	✗	✓
vdW/h	V483	R_H50	✓	✓	✓	✓	✓	✓	✓	✓
vdW/h	V483	N_H59	✓	✓	✓	✓	✓	✓	✓	✓
vdW/h	V483	T_L94	✓	✓	✓	✓	✓	✓	✓	✓
vdW/h	V483	R_L96	✓	✓	✓	✓	✓	✓	✓	✗
π/π	F486	Y_L32	✓	✓	✓	✓	✓	✓	✗(vdW/h)	✓
π/π	F486	Y_L92	✓	✓	✓	✓	✓	✓	✗(vdW/h)	✓
vdW/h	Y489	Y_H110	✓	✓	✓	✓	✗	✓	✓	✓
p	Y489	Y_L32	✓	✓	✗	✗	✗	✗	✗	✓

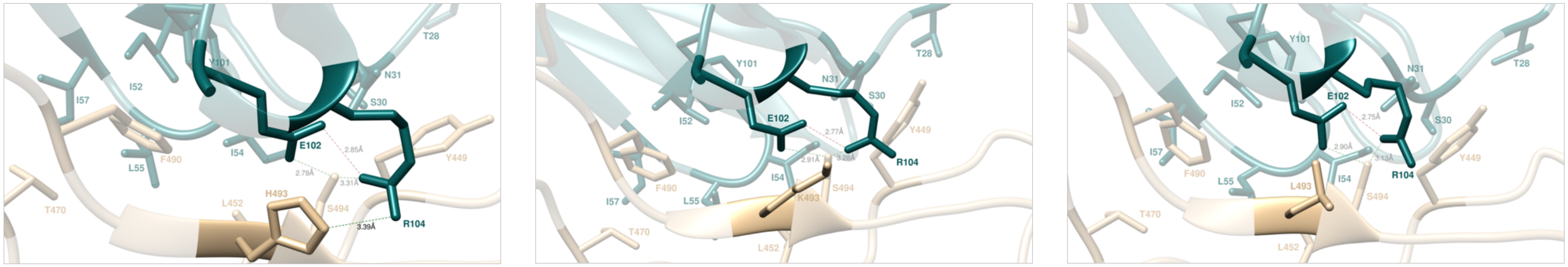

Fig. S2. Main interactions involving the S-RBD$_{CoV-2}$ H493 (left), K493 (middle), and L493 (right) at the interface with the LY-CoV555 antibody as obtained from the corresponding equilibrated MD simulations. The wild type Q493 and the R493 mutant complexes are presented and discussed in the main text (Figures 2E and 4B). Colors and other explanations as in Figure S1. For further details see Tables S1 and S6.

Table S6. Main interactions between the wild-type S-RBD$_{CoV-2}$ residue Q493 and all considered mutants* at the protein-protein interface detected during MD simulations of RBD of SARS-CoV-2 (COV2) in complex with the LY-CoV555 antibody. Acronyms and other explanations as in Table S5. *Mutant R493 is discussed in detail in main text.

**HB**	**COV2**	**LY-CoV555**	**Q493**	**H493**	**K493**	**L493**	**R493**
s-s	X493	E_H102	✓(3.25)	✗	✗	✗(vdW/h)	✗(vdW/h)
s-s	X493	R_H104	✓(3.31)	✓(3.39)	✗(vdW/h)	✗(vdW/h)	✗(vdW/h)
s-b	X493	R_H104	✓(3.34)	✗(π/c)	✗	✗	✗
**HB**	**COV2**	**LY-CoV555**	**Q493**	**H493**	**K493**	**L493**	**R493**
s-s	S494	E_H102	✓(2.86)	✓(2.79)	✓(2.91)	✓(2.90)	✓(2.59)
s-s	S494	R_H104	✓(3.18)	✓(3.31)	✓(3.28)	✓(3.13)	✓(3.24)
**CI**	**COV2**	**LY-CoV555**	**Q493**	**H493**	**K493**	**L493**	**R493**
p	Y449	T_H28	✓	✗	✗	✗	✗
p	Y449	S_H30	✓	✓	✗	✗	✗(vdW/h)
p	Y449	N_H31	✓	✓	✓	✓	✓
vdW/h	Y449	I_H54	✓	✓	✓	✓	✓

vdW/h	L452	I_H54	√	√	√	√	√
vdW/h	L452	L_H55	√	√	√	√	√
vdW/h	T470	L_H55	√	√	√	√	√
vdW/h	T470	I_H57	√	√	√	√	√
vdW/h	F490	I_H52	√	√	√	√	√
vdW/h	F490	L_H55	√	√	√	√	√
vdW/h	F490	I_H57	√	√	√	√	√
π/π	F490	Y_H101	√	√	√	√	√
p	S494	N_H31	√	√	√	√	√
**SB**	**LY-CoV555**	**LY-CoV555**	**Q493**	**H493**	**K493**	**L493**	**R493**
s-s	E_H102	R_H104	√(2.76)	√(2.85)	√(2.77)	√(2.75)	√(2.69)

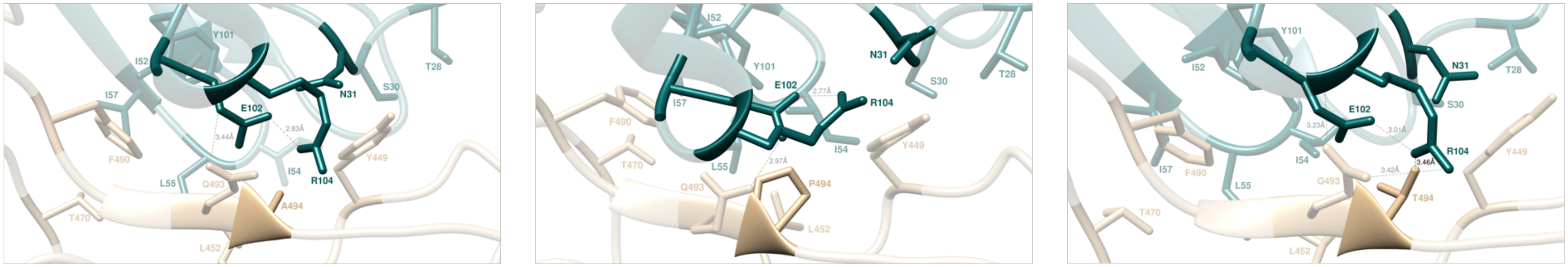

Fig. S3. Main interactions involving the S-RBD$_{CoV-2}$ A494 (left), P494 (middle), and T494 (right) at the interface with the LY-CoV555 antibody as obtained from the corresponding equilibrated MD simulations. The wild type S494 and the R494 mutant complexes are presented and discussed in the main text (Figures 2E and 4C). Colors and other explanations as in Figure S1. For further details see Tables S1 and S7.

Table S7. Main interactions between the wild-type S-RBD$_{CoV-2}$ residue S494 and all considered mutants* at the protein-protein interface detected during MD simulations of RBD of SARS-CoV-2 (COV2) in complex with the LY-CoV555 antibody. Acronyms and other explanations as in Table S5. *Mutant R494 is discussed in detail in main text.

**HB**	**COV2**	**LY-CoV555**	**S494**	**A494**	**P494**	**R494**	**T494**
s-s	X494	E_H102	✓(2.86)	✗	✗	✓(SB,2.76)	✗(p)
s-s	X494	R_H104	✓(3.18)	✗	✗	✗	✓(3.46)
**CI**	**COV2**	**LY-CoV555**	**S494**	**A494**	**P494**	**R494**	**T494**
p	X494	N_H31	✓	✗	✗	✓	✓
**HB**	**COV2**	**LY-CoV555**	**S494**	**A494**	**P494**	**R494**	**T494**
s-s	Q493	E_H102	✓(3.25)	✗(p)	✓(2.97)	✗(p)	✗(p)
s-s	Q493	R_H104	✓(3.31)	✗(p)	✗	✗(p)	✓(3.42)
s-b	Q493	R_H104	✓(3.34)	✓(3.44)	✗	✗	✓(3.23)
**CI**	**COV2**	**LY-CoV555**	**S494**	**A494**	**P494**	**R494**	**T494**
p	Y449	T_H28	✓	✗	✗	✗	✓
p	Y449	S_H30	✓	✗	✓	✗	✓

p	Y449	N_H31	✓	✓	✓	✗	✓
vdW/h	Y449	I_H54	✓	✓	✓	✗	✓
vdW/h	L452	I_H54	✓	✓	✓	✓	✓
vdW/h	L452	L_H55	✓	✓	✓	✓	✓
vdW/h	T470	L_H55	✓	✓	✓	✓	✓
vdW/h	T470	I_H57	✓	✓	✓	✓	✓
vdW/h	F490	I_H52	✓	✓	✓	✓	✓
vdW/h	F490	L_H55	✓	✓	✓	✓	✓
vdW/h	F490	I_H57	✓	✓	✓	✓	✓
π-π	F490	Y_H101	✓	✓	✓	✓	✓
**SB**	**LY-CoV555**	**LY-CoV555**	**S494**	**A494**	**P494**	**R494**	**T494**
s-s	E_H102	R_H104	✓(2.76)	✓(2.83)	✓(2.77)	✓(2.81)	✓(3.01)

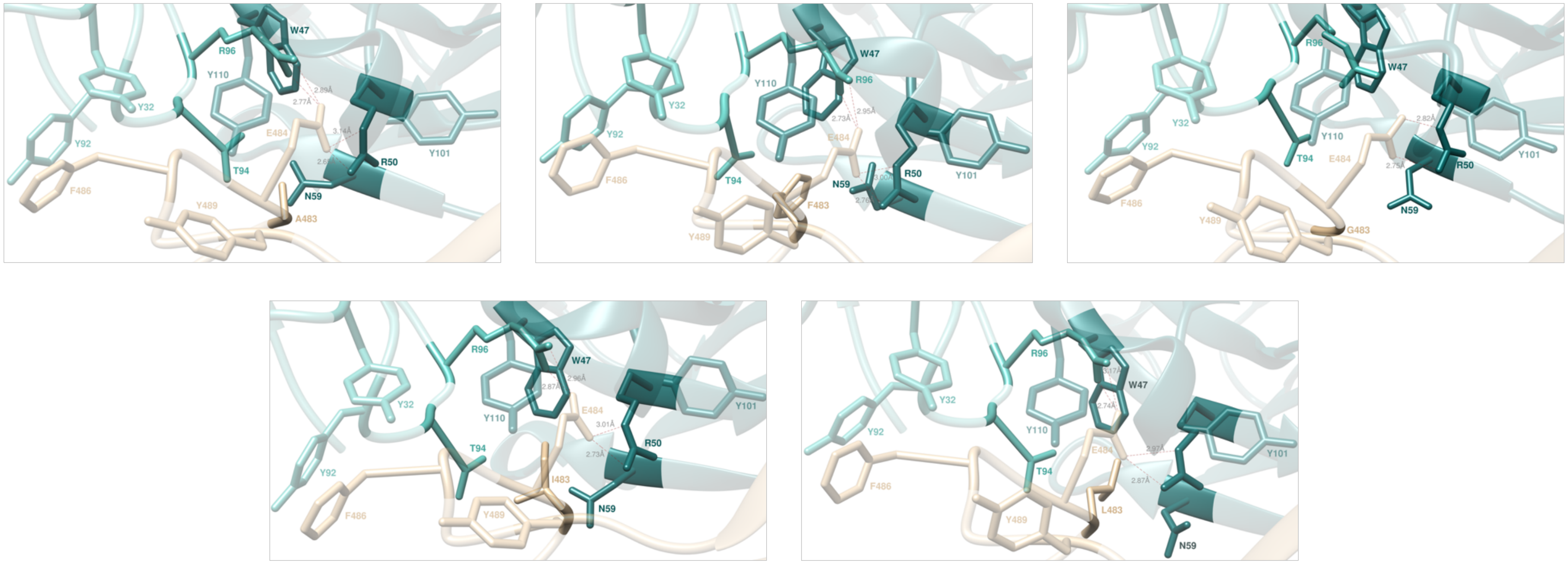

Fig. S4. Main interactions involving the S-RBD$_{CoV-2}$ A483 (top left), F483 (top middle), G483 (top right), I483 (bottom left), and L483 (bottom right) at the interface with the LY-CoV555 antibody as obtained from the corresponding equilibrated MD simulations. The wild type V483 is presented and discussed in the main text (Figures 2D). Colors and other explanations as in Figure S1. For further details see Tables S1 and S8.

Table S8. Main interactions between the wild-type S-RBD$_{CoV-2}$ residue V483 and all considered mutants at the protein-protein interface detected during MD simulations of RBD of SARS-CoV-2 (COV2) in complex with the LY-CoV555 antibody. Acronyms and other explanations as in Table S5.

**SB**	**COV2**	**LY-CoV555**	**V483**	**A483**	**F483**	**G483**	**I483**	**L483**
s-s	E484	R_H50	✓(2.74,3.05)	✓(2.65,3.14)	✓(2.76,3.00)	✓(2.75,2.82)	✓(2.73,3.01)	✓(2.87,2.97)
s-s	E484	R_L96	✓(2.82,2.96)	✓(2.77,2.89)	✓(2.73,2.95)	✗	✓(2.87,2.96)	✓(2.74,3.17)
**CI**	**COV2**	**LY-CoV555**	**V483**	**A483**	**F483**	**G483**	**I483**	**L483**
vdW/h	E484	Y_H101	✓	✓	✓	✓	✓	✓

vdW/h	E484	Y_H110	✓	✓	✓	✓	✓	✓
**CI**	**COV2**	**LY-CoV555**	**V483**	**A483**	**F483**	**G483**	**I483**	**L483**
s-s	X483	W_H47	✓	✓	✓	✗	✓	✓
s-s	X483	R_H50	✓	✗	✓	✗	✓	✓
s-s	X483	N_H59	✓	✗	✓	✗	✓	✓
s-s	X483	T_L94	✓	✗	✓	✗	✓	✓
s-s	X483	R_L96	✓	✓	✓	✗	✓	✓
π-π	F486	Y_L32	✓	✓	✓	✓	✓	✓
π-π	F486	Y_L92	✓	✓	✓	✓	✓	✓
vdW/h	Y489	Y_H110	✓	✓	✓	✓	✓	✓
p	Y489	Y_L32	✓	✓	✓	✓	✓	✓

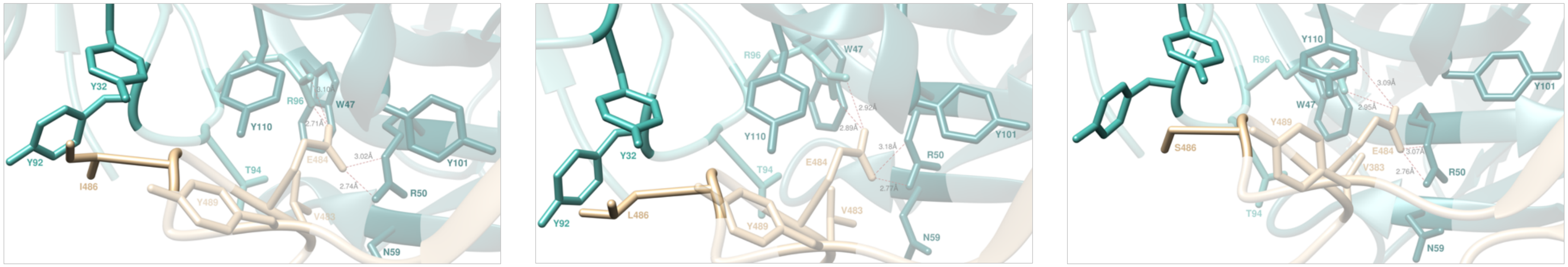

Fig. S5. Main interactions involving the S-RBD$_{CoV-2}$ I486 (left), L486 (middle), and S486 (right) at the interface with the LY-CoV555 antibody as obtained from the corresponding equilibrated MD simulations. The wild type F486 is presented and discussed in the main text (Figures 2D). Colors and other explanations as in Figure S1. For further details see Tables S1 and S9.

Table S9. Main interactions between the wild-type S-RBD$_{CoV-2}$ residue V483 and all considered mutants at the protein-protein interface detected during MD simulations of RBD of SARS-CoV-2 (COV2) in complex with the LY-CoV555 antibody. Acronyms and other explanations as in Table S5.

**SB**	**COV2**	**LY-CoV555**	**F486**	**I486**	**L486**	**S486**
s-s	E484	R_H50	✓(2.74,3.05)	✓(2.74,3.02)	✓(2.77,3.18)	✓(2.76,3.07)
s-s	E484	R_L96	✓(2.82,2.96)	✓(2.71,3.10)	✓(2.89,2.92)	✓(2.95,3.09)
**CI**	**COV2**	**LY-CoV555**	**F486**	**I486**	**L486**	**S486**
vdW/h	E484	Y_H101	✓	✓	✓	✓
vdW/h	E484	Y_H110	✓	✓	✓	✓
**CI**	**COV2**	**LY-CoV555**	**F486**	**I486**	**L486**	**S486**
s-s	V483	W_H47	✓	✓	✓	✓
s-s	V483	R_H50	✓	✓	✓	✓
s-s	V483	N_H59	✓	✓	✓	✓
s-s	V483	T_L94	✓	✓	✓	✓
s-s	V483	R_L96	✓	✓	✓	✓
π-π	X486	Y_L32	✓	✗(vdw)	✗(vdw)	✗

π-π	X486	Y_L92	✓	✗(vdw)	✗(vdw)	✗(vdw)
vdW/h	Y489	Y_H110	✓	✓	✓	✓
p	Y489	Y_L32	✓	✓	✓	✓

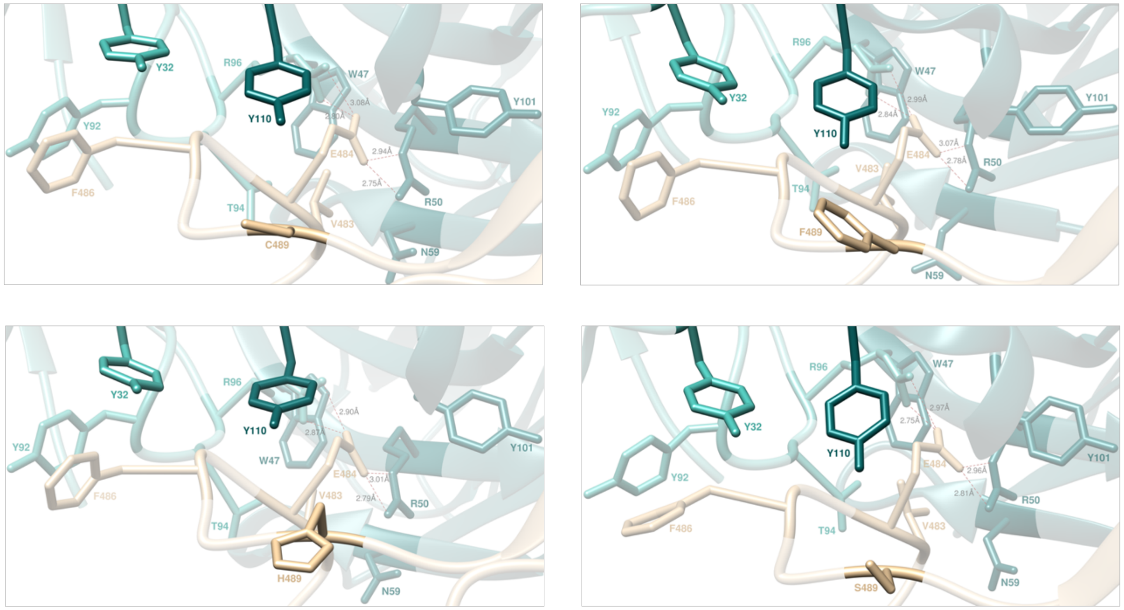

Fig. S6. Main interactions involving the S-RBD$_{CoV-2}$ C489 (top left), F489 (top right), H489 (bottom left), and S486 (bottom right) at the interface with the LY-CoV555 antibody as obtained from the corresponding equilibrated MD simulations. The wild type Y489 is presented and discussed in the main text (Figures 2D). Colors and other explanations as in Figure S1. For further details see Tables S1 and S10.

Table S10. Main interactions between the wild-type S-RBD$_{CoV-2}$ residue Y489 and all considered mutants at the protein-protein interface detected during MD simulations of RBD of SARS-CoV-2 (COV2) in complex with the LY-CoV555 antibody. Acronyms and other explanations as in Table S5.

**SB**	**COV2**	**LY-CoV555**	**Y489**	**C489**	**F489**	**H489**	**S489**
s-s	E484	R_H50	✓(2.74,3.05)	✓(2.75,2.94)	✓(2.78,3.07)	✓(2.79,3.01)	✓(2.81,2.96)
s-s	E484	R_L96	✓(2.82,2.96)	✓(2.80,3.08)	✓(2.84,2.99)	✓(2.87,2.90)	✓(2.75,2.97)
**CI**	**COV2**	**LY-CoV555**	**Y489**	**C489**	**F489**	**H489**	**S489**
vdW/h	E484	Y_H101	✓	✓	✓	✓	✓
vdW/h	E484	Y_H110	✓	✓	✓	✓	✓

CI	COV2	LY-CoV555	Y489	C489	F489	H489	S489
s-s	V483	W_H47	✓	✓	✓	✓	✓
s-s	V483	R_H50	✓	✓	✓	✓	✓
s-s	V483	N_H59	✓	✓	✓	✓	✓
s-s	V483	T_L94	✓	✓	✓	✓	✓
s-s	V483	R_L96	✓	✓	✓	✓	✓
π-π	F486	Y_L32	✓	✓	✓	✓	✓
π-π	F486	Y_L92	✓	✓	✓	✓	✓
vdW/h	X489	Y_H110	✓	✓	✓	✓	✓
p	X489	Y_L32	✓	✗	✗	✗	✗

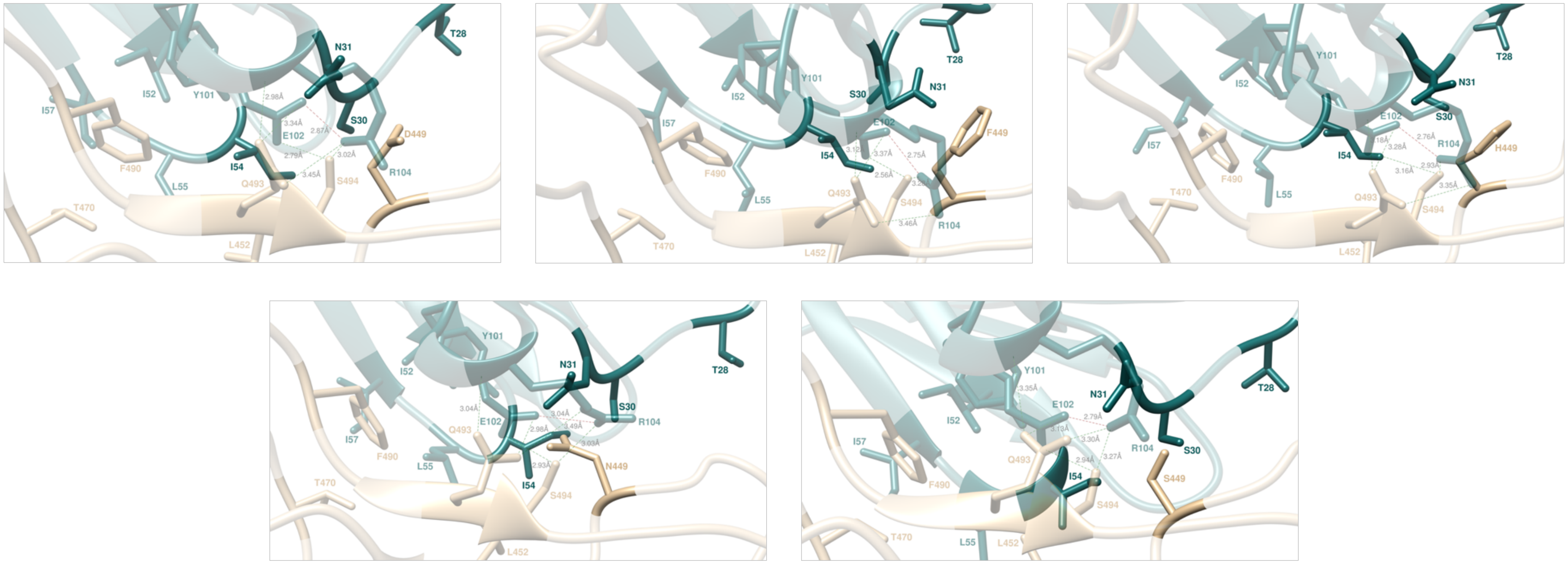

Fig. S7. Main interactions involving the S-RBD$_{CoV-2}$ D449 (top left), F449 (top middle), H449 (top right), N449 (bottom left), and S449 (bottom right) at the interface with the LY-CoV555 antibody as obtained from the corresponding equilibrated MD simulations. The wild-type Y449 is presented and discussed in the main text (Figure 2E). For further details see Tables S1 and S11.

Table S11. Main interactions between the wild-type S-RBD$_{CoV-2}$ residue Y449 and all considered mutants at the protein-protein interface detected during MD simulations of the RBD of SARS-CoV-2 (COV2) in complex with the LY-CoV555 antibody. Acronyms and other explanations as in Table S5.

HB	COV2	LY-CoV555	Y449	D449	F449	H449	N449	S449
s-s	Q493	E_H102	✓(3.25)	✓(3.34)	✓(3.37)	✓(3.28)	✓(2.98)	✓(3.13)
s-s	Q493	R_H104	✓(3.31)	✓(3.45)	✓(3.46)	✓(3.35)	✓(3.49)	✓(3.30)
s-b	Q493	R_H104	✓(3.34)	✓(2.98)	✓(3.12)	✓(3.18)	✓(3.04)	✓(3.35)
**HB**	**COV2**	**LY-CoV555**	**Y449**	**D449**	**F449**	**H449**	**N449**	**S449**
s-s	S494	E_H102	✓(2.86)	✓(2.79)	✓(2.56)	✓(3.16)	✓(2.93)	✓(2.94)

s-s	S494	R_H104	✓(3.18)	✓(3.02)	✓(3.28)	✓(2.93)	✓(3.03)	✓(3.27)
**CI**	**COV-2**	**LY-CoV555**	**Y449**	**D449**	**F449**	**H449**	**N449**	**S449**
p	X449	T_H28	✓	✗	✗(vdW/h)	✓	✗	✗
p	X449	S_H30	✓	✓	✗	✓	✓	✓
p	X449	N_H31	✓	✓	✗(vdW/h)	✓	✓	✓
vdW/h	X449	I_H54	✓	✗	✓	✓	✓	✗
vdW/h	L452	I_H54	✓	✓	✓	✓	✓	✓
vdW/h	L452	L_H55	✓	✓	✓	✓	✓	✓
vdW/h	T470	L_H55	✓	✓	✓	✓	✓	✓
vdW/h	T470	I_H57	✓	✓	✓	✓	✓	✓
vdW/h	F490	I_H52	✓	✓	✓	✓	✓	✓
vdW/h	F490	L_H55	✓	✓	✓	✓	✓	✓
vdW/h	F490	I_H57	✓	✓	✓	✓	✓	✓
π-π	F490	Y_H101	✓	✓	✓	✓	✓	✓
p	S494	N_H31	✓	✓	✓	✓	✓	✓
**SB**	**LY-CoV555**	**LY-CoV555**	**Y449**	**D449**	**F449**	**H449**	**N449**	**S449**
s-s	E_H102	R_H104	✓(2.76)	✓(2.87)	✓(2.75)	✓(2.76)	✓(3.04)	✓(2.79)

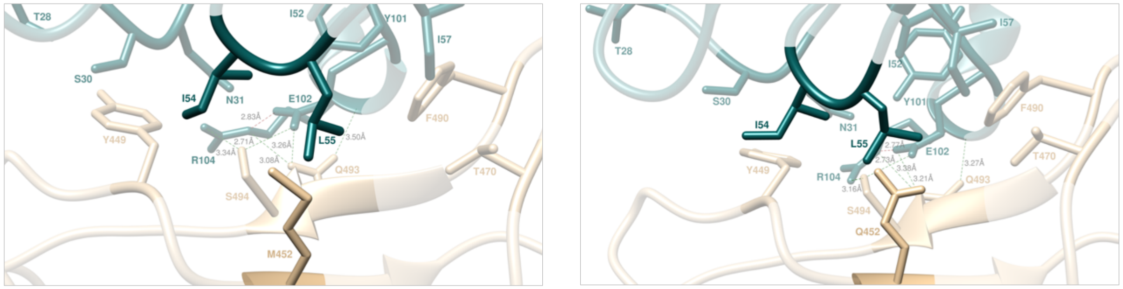

Fig. S8. Main interactions involving the S-RBD$_{CoV-2}$ M452 (left) and Q452 (right) at the interface with the LY-CoV555 antibody as obtained from the corresponding equilibrated MD simulations. The wild type L452 and the R452 mutant complexes are presented and discussed in the main text (Figures 2E and 4D). Colors and other explanations as in Figure S1. For further details see Tables S1 and S12.

Table S12. Main interactions between the wild-type S-RBD$_{CoV-2}$ residue L452 and all considered mutants* at the protein-protein interface detected during MD simulations of RBD of SARS-CoV-2 (COV2) in complex with the LY-CoV555. Acronyms and other explanations as in Table S5. *Mutant R452 is discussed in detail in main text.

**SB**	**COV2**	**LY-CoV555**	**L452**	**M452**	**Q452**	**R452**
s-s	X452	E_H102	✗	✗	✗	✓(3.15)
**HB**	**COV2**	**LY-CoV555**	**L452**	**M452**	**Q452**	**R452**
s-s	Q493	E_H102	✓(3.25)	✓(3.26)	✓(3.21)	✗
s-s	Q493	R_H104	✓(3.31)	✓(3.08)	✓(3.38)	✗(p)
s-b	Q493	R_H104	✓(3.34)	✓(3.50)	✓(3.27)	✓(2.87)
**HB**	**COV2**	**LY-CoV555**	**L452**	**M452**	**Q452**	**R452**
s-s	S494	E_H102	✓(2.86)	✓(2.71)	✓(2.73)	✗(p)
s-s	S494	R_H104	✓(3.18)	✓(3.34)	✓(3.16)	✗
**CI**	**COV2**	**LY-CoV555**	**L452**	**M452**	**Q452**	**R452**
p	Y449	T_H28	✓	✓	✓	✗
p	Y449	S_H30	✓	✓	✓	✗

p	Y449	N_H31	✓	✓	✓	✓
vdW/h	Y449	I_H54	✓	✓	✓	✗
vdW/h	X452	I_H54	✓	✓	✓	✓
vdW/h	X452	L_H55	✓	✓	✓	✗
vdW/h	T470	L_H55	✓	✓	✓	✓
vdW/h	T470	I_H57	✓	✓	✓	✓
vdW/h	F490	I_H52	✓	✓	✓	✓
vdW/h	F490	L_H55	✓	✓	✓	✓
vdW/h	F490	I_H57	✓	✓	✓	✓
π-π	F490	Y_H101	✓	✓	✓	✓
p	S494	N_H31	✓	✓	✓	✗
**SB**	**LY-CoV555**	**LY-CoV555**	**L452**	**M452**	**Q452**	**R452**
s-s	E_H102	R_H104	✓(2.76)	✓(2.83)	✓(2.77)	✗

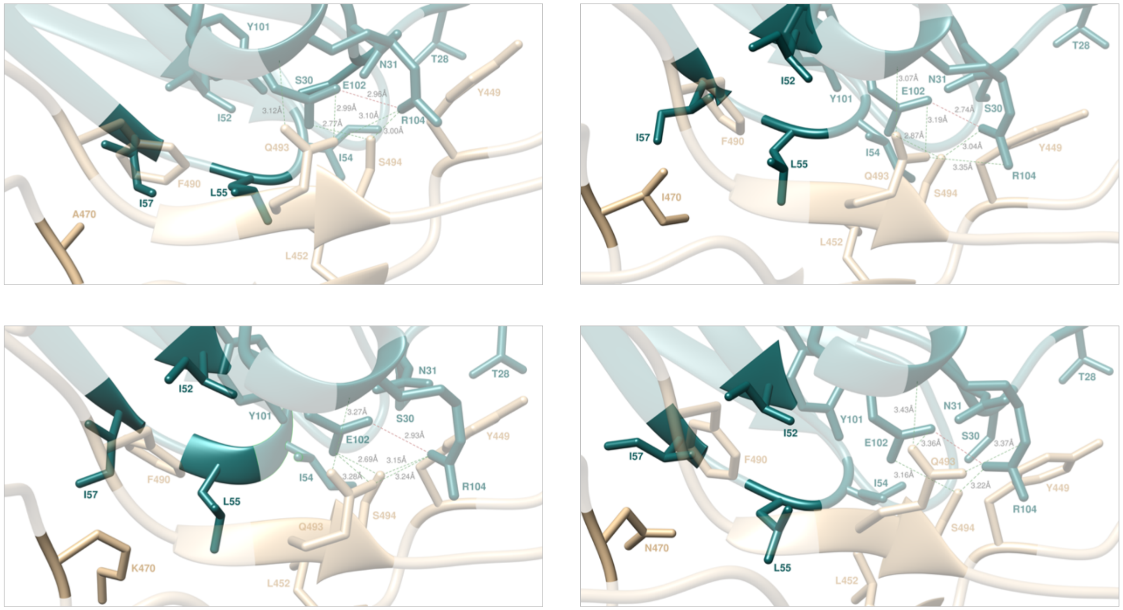

Fig. S9. Main interactions involving the S-RBD$_{CoV-2}$ A470 (top left), I470 (top right), K470 (bottom left), and N470 (bottom right) at the interface with the LY-CoV555 antibody as obtained from the corresponding equilibrated MD simulations. The wild-type T470 is presented and discussed in the main text (Figure 2E). For further details see Tables S1 and S13.

Table S13. Main interactions between the wild-type S-RBD$_{CoV-2}$ residue T470 and all considered mutants at the protein-protein interface detected during MD simulations of the RBD of SARS-CoV-2 (COV2) in complex with the LY-CoV555 antibody.

HB	COV2	LY-CoV555	T470	A470	I470	K470	N470
s-s	Q493	E_H102	✓(3.25)	✓(2.99)	✓(3.19)	✓(3.28)	✓(3.36)
s-s	Q493	R_H104	✓(3.31)	✓(3.16)	✓(3.35)	✓(3.24)	✓(3.37)
s-b	Q493	R_H104	✓(3.34)	✓(3.12)	✓(3.07)	✓(3.27)	✓(3.43)
**HB**	**COV2**	**LY-CoV555**	**T470**	**A470**	**I470**	**K470**	**N470**

s-s	S494	E_H102	√(2.86)	√(2.77)	√(2.87)	√(2.69)	√(3.16)
s-s	S494	R_H104	√(3.18)	√(3.00)	√(3.04)	√(3.15)	√(3.22)
**CI**	**COV2**	**LY-CoV555**	**T470**	**A470**	**I470**	**K470**	**N470**
p	Y449	T_H28	√	√	√	√	√
p	Y449	S_H30	√	√	√	√	√
p	Y449	N_H31	√	√	√	√	√
vdW/h	Y449	I_H54	√	√	√	√	√
vdW/h	L452	I_H54	√	√	√	√	√
vdW/h	L452	L_H55	√	√	√	√	√
vdW/h	X470	L_H55	√	✗	√	√	√
vdW/h	X470	I_H57	√	√	√	√	√
vdW/h	F490	I_H52	√	√	√	√	√
vdW/h	F490	L_H55	√	√	√	√	√
vdW/h	F490	I_H57	√	√	√	√	√
π-π	F490	Y_H101	√	√	√	√	√
p	S494	N_H31	√	√	√	√	√
**SB**	**LY-CoV555**	**LY-CoV555**	**T470**	**A470**	**I470**	**K470**	**N470**
s-s	E_H102	R_H104	√(2.76)	√(2.96)	√(2.74)	√(2.93)	√(2.76)

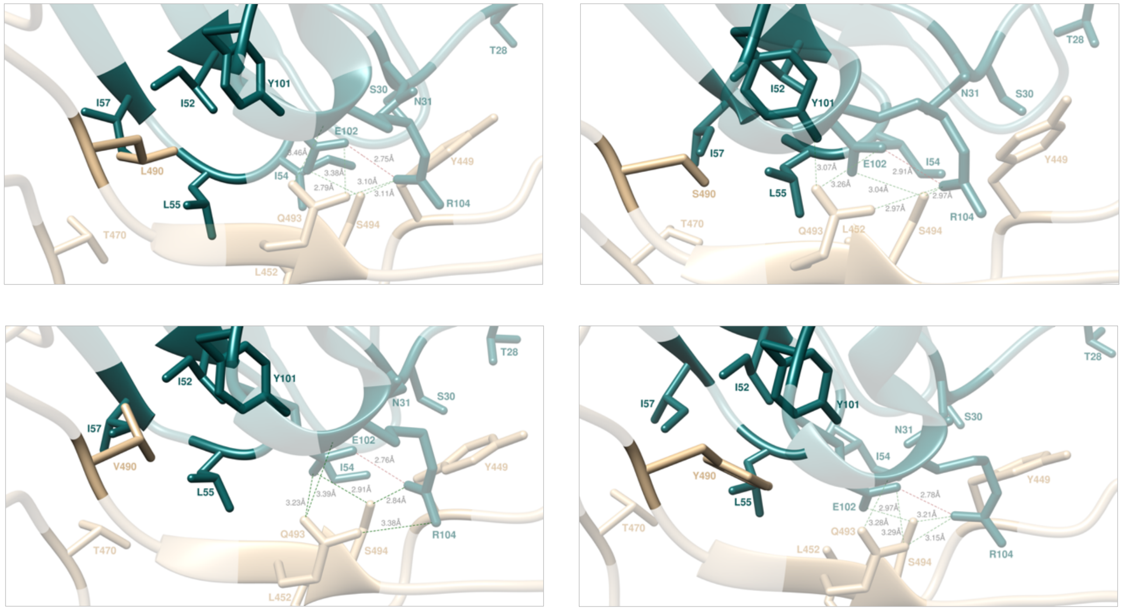

Fig. S10. Main interactions involving the S-RBD$_{CoV-2}$ L490 (top left), S490 (top right), V490 (bottom left), and Y490 (bottom right) at the interface with the LY-CoV555 antibody as obtained from the corresponding equilibrated MD simulations. The wild-type F490 is presented and discussed in the main text (Figure 2E). For further details see Tables S1 and S13.

Table S14. Main interactions between the wild-type S-RBD$_{CoV-2}$ residue F490 and all considered mutants at the protein-protein interface detected during MD simulations of the RBD of SARS-CoV-2 (COV2) in complex with the LY-CoV555 antibody.

HB	COV2	LY-CoV555	F490	L490	S490	V490	Y490
s-s	Q493	E_H102	✓(3.25)	✓(3.38)	✓(3.26)	✓(3.23)	✓(3.29)
s-s	Q493	R_H104	✓(3.31)	✓(3.10)	✓(2.97)	✓(3.38)	✓(3.15)
s-b	Q493	R_H104	✓(3.34)	✓(3.46)	✓(3.07)	✓(3.39)	✓(3.28)
**HB**	**COV2**	**LY-CoV555**	**F490**	**L490**	**S490**	**V490**	**Y490**

s-s	S494	E_H102	√(2.86)	√(2.79)	√(3.94)	√(2.91)	√(2.97)
s-s	S494	R_H104	√(3.18)	√(3.11)	√(2.97)	√(2.84)	√(3.21)
**CI**	**COV2**	**LY-CoV555**	**F490**	**L490**	**S490**	**V490**	**Y490**
p	Y449	T_H28	√	√	√	√	√
p	Y449	S_H30	√	√	√	√	√
p	Y449	N_H31	√	√	√	√	√
vdW/h	Y449	I_H54	√	√	√	√	√
vdW/h	L452	I_H54	√	√	√	√	√
vdW/h	L452	L_H55	√	√	√	√	√
vdW/h	T470	L_H55	√	√	√	√	√
vdW/h	T470	I_H57	√	√	√	√	√
vdW/h	X490	I_H52	√	√	✗	√	√
vdW/h	X490	L_H55	√	√	√	√	√
vdW/h	X490	I_H57	√	✗	✗	✗	√
π-π	X490	Y_H101	√	✗(vdW)	✗	✗	√
p	S494	N_H31	√	√	√	√	√
**SB**	**LY-CoV555**	**LY-CoV555**	**F490**	**L490**	**S490**	**V490**	**Y490**
s-s	E_H102	R_H104	√(2.76)	√(2.75)	√(2.91)	√(2.76)	√(2.78)

Table S15. Main intermolecular and intramolecular interactions between residues at the protein-protein interface detected during MD simulations of the LY-Cov016 antibody in complex with the RBDs of SARS-CoV-2 S-RBD (COV2). HB = hydrogen bond; SB = salt bridge; CI = contact interactions, including van der Waals/hydrophobic (vdW/h), polar (p), π/π and π/cation (π/c) interactions. In the HB column, s-s indicates side chain-side chain interactions while s-b or b-s indicate side chain-backbone or backbone-sidechain interactions, respectively.

HB	COV2	Length (Å)	LY-Cov016
s-s	E406	2.84 ± 0.27	T_L94
s-s	T415	3.13 ± 0.10	S_H56
s-s	K417	2.98 ± 0.14	Y_H52
s-s	D420	3.07 ± 0.15	S_H56
s-s<br>s-b	Y421	3.09 ± 0.11<br>3.21 ± 0.14	S_H53<br>G_H54
s-b<br>s-s	N460	3.12 ± 0.17<br>3.04 ± 0.09	G_H54<br>S_H56
s-s	Y473	2.83 ± 0.21	S_H31
s-s	N487	2.86 ± 0.15	R_H97
s-s	Y489	3.38 ± 0.13	R_H97
s-b	Q493	3.03 ± 0.18	Y_H102
s-s<br>b-s	Y505	3.01 ± 0.19<br>3.14 ± 0.13	Y_L32<br>Y_L92
**SB**	**COV2**	**Length (Å)**	**LY-Cov016**
	K417	2.92 ± 0.15<br>3.10 ± 0.17	D_H104

CI	COV2	LY-Cov016
p<br>vdW/h	K417	Y_H33<br>P_H100
p<br>vdW/h	Y421	Y_H33<br>Y_H52

vdW/h		Y_H33
vdW/h	L455	P_H100
vdW/h		M_H101
vdW/h		Y_H33
vdW/h		S_H53
vdW/h	F456	L_H99
vdW/h		P_H100
vdW/h		M_H101
p	Y473	S_H53
vdW/h	N487	F_H27
vdW/h	Y489	L_H99
vdW/h		M_H101
vdW/h	Q493	M_H101
vdW/h		Y_H102

Table S16. Relative binding free energy and its components calculated by the combined computational alanine scanning mutagenesis – interaction entropy approach for the S-RBD of SARS-CoV-2 residues effectively involved in the binding interface with the LY-CoV016 antibody (see the SI Materials and Methods section for details). IE = interaction entropy. $\Delta\Delta G = \Delta G_{WILDTYPE} - \Delta G_{ALA}$ (see text for details).

	**E406A**	**T415A**	**K417A**	**D420A**	**Y421A**	**L455A**	**F456A**	**N460A**	**Y473A**	**N487A**
$\Delta\Delta E_{DISP}$	-0.25	0.08	-0.81	-0.30	-1.04	-1.37	-2.62	-1.21	-0.44	-0.47
$\Delta\Delta E_{ELE}$	-0.87	-1.26	-5.43	-1.87	-2.59	0.22	0.22	-1.40	-1.78	-1.62
$\Delta\Delta H$	-1.12	-1.18	-6.24	-2.17	-3.63	-1.15	-2.40	-2.61	-2.22	-2.09
$\Delta\Delta IE$	-0.17	0.02	0.23	0.16	0.16	-0.05	-0.09	-0.14	0.14	0.06
$\mathbf{\Delta\Delta G_{ACE2}}$	**-1.29 (0.13)**	**-1.16 (0.14)**	**-6.01 (0.10)**	**-2.01 (0.11)**	**-2.47 (0.18)**	**-1.20 (0.16)**	**-2.49 (0.13)**	**-2.75 (0.11)**	**-2.08 (0.12)**	**-2.03 (0.16)**

	**Y489A**	**Q493A**	**Y505A**
$\Delta\Delta E_{DISP}$	-1.49	-0.58	-0.83
$\Delta\Delta E_{ELE}$	-0.58	-0.92	-1.23
$\Delta\Delta H$	-2.07	-1.50	-2.06
$\Delta\Delta IE$	0.23	-0.09	0.19
$\mathbf{\Delta\Delta G_{ACE2}}$	**-1.84 (0.09)**	**-1.59 (0.14)**	**-1.87 (0.12)**

Table S17. Relative binding free energy and its components calculated by the combined computational alanine scanning mutagenesis – interaction entropy approach for LY-Cov016 antibody residues effectively involved in the binding interface with the S-RBD of SARS-CoV-2 (see the SI Materials and Methods section for details). IE = interaction entropy. $\Delta\Delta G = \Delta G_{WILDTYPE} - \Delta G_{ALA}$ (see text for details).

	**S_H31A**	**Y_H33A**	**Y_H52A**	**S_H53A**	**S_H56A**	**R_H97A**	**L_H99A**	**P_H100A**	**M_H101A**	**Y_H102A**
$\Delta\Delta E_{DISP}$	-0.03	-0.44	-0.88	-1.05	-1.49	-0.27	-0.92	-1.22	-1.89	-0.50
$\Delta\Delta E_{ELE}$	-0.88	-2.01	-0.83	-1.47	-2.40	-1.99	0.08	0.12	0.04	-1.32
$\Delta\Delta H$	-0.91	-2.45	-1.71	-2.52	-3.89	-2.26	-0.84	-1.10	-1.85	-1.82
$\Delta\Delta IE$	-0.07	0.08	0.16	-0.04	0.09	0.23	-0.05	-0.17	0.07	0.19
**$\Delta\Delta G_{CoV-2}$**	**-0.98 (0.17)**	**-2.37 (0.12)**	**-1.55 (0.11)**	**-2.56 (0.15)**	**-3.80 (0.16)**	**-2.03 (0.08)**	**-0.89 (0.15)**	**-1.27 (0.10)**	**-1.78 (0.14)**	**-1.63 (0.18)**

	**D_H104A**	**Y_L32A**	**Y_L92A**	**T_L94A**
$\Delta\Delta E_{DISP}$	-0.18	-0.33	-0.28	0.16
$\Delta\Delta E_{ELE}$	-2.84	-0.92	-0.86	-1.15
$\Delta\Delta H$	-3.02	-1.25	-1.14	-0.99
$\Delta\Delta IE$	0.21	0.13	0.15	-0.16
**$\Delta\Delta G_{CoV-2}$**	**-2.81 (0.15)**	**-1.12 (0.10)**	**-0.99 (0.16)**	**-1.15 (0.12)**

Table S18. Relative binding free energy and its components for the mutated S-RBD of SARS-CoV-2 residues in the binding interface with the LY-Cov016 antibody. IE = interaction entropy. IE = interaction entropy. $\Delta\Delta G = \Delta G_{WILDTYPE} - \Delta G_{MUTANT}$ (see text for details).

	**E406D**	**E406Q**	**T415A**	**T415I**	**T415N**	**T415P**	**T415S**	**K417E**	**K417N**	**K417R**	**K417T**	**D420A**
$\Delta\Delta E_{DISP}$	-0.23	-0.18	-0.59	-0.37	-0.61	-1.32	-0.32	-2.76	-2.37	-0.31	-2.99	-1.89
$\Delta\Delta E_{ELE}$	0.14	-0.11	-1.34	-1.21	-0.70	-1.64	-0.03	-4.64	-4.75	-0.80	-4.12	-2.61
$\Delta\Delta H$	-0.09	-0.29	-1.93	-1.58	-1.31	-2.96	-0.35	-7.40	-7.13	-1.11	-7.11	-4.50
$\Delta\Delta IE$	0.02	0.07	0.06	-0.07	0.10	0.13	-0.05	-0.16	-0.15	0.12	-0.03	0.14
$\boldsymbol{\Delta\Delta G_{CoV2}}$	**-0.07**	**-0.22**	**-1.87**	**-1.65**	**-1.21**	**-2.83**	**-0.40**	**-7.56**	**-7.27**	**-0.99**	**-7.14**	**-4.36**
	**(0.18)**	**(0.16)**	**(0.14)**	**(0.18)**	**(0.17)**	**(0.07)**	**(0.13)**	**(0.18)**	**(0.07)**	**(0.15)**	**(0.09)**	**(0.17)**
	**D420G**	**D420N**	**L455F**	**L455S**	**L455V**	**F456L**	**F456Y**	**N460I**	**N460K**	**N460S**	**N460T**	**Y473F**
$\Delta\Delta E_{DISP}$	-2.06	-2.72	0.15	-1.07	-0.83	-0.58	-0.19	-1.97	-1.70	-2.35	-1.94	-0.33
$\Delta\Delta E_{ELE}$	-2.54	-1.45	-0.22	0.16	0.17	-0.09	-0.03	-3.03	-1.37	-1.84	-2.06	-1.50
$\Delta\Delta H$	-4.60	-4.17	-0.07	-0.91	-0.66	-0.67	-0.22	-5.00	-3.07	-4.19	-4.00	-1.83
$\Delta\Delta IE$	0.21	-0.06	-0.11	0.08	0.07	0.06	0.18	-0.01	-0.21	0.13	-0.12	0.16
$\boldsymbol{\Delta\Delta G_{CoV-2}}$	**-4.39**	**-4.23**	**-0.18**	**-0.83**	**-0.59**	**-0.61**	**-0.04**	**-5.01**	**-3.28**	**-4.06**	**-4.12**	**-1.67**
	**(0.10)**	**(0.10)**	**(0.17)**	**(0.11)**	**(0.14)**	**(0.16)**	**(0.17)**	**(0.14)**	**(0.18)**	**(0.09)**	**(0.14)**	**(0.08)**
	**Y473H**	**N487D**	**Y489C**	**Y489F**	**Y489H**	**Y489S**	**Q493H**	**Q493K**	**Q493L**	**Q493R**	**Y505F**	**Y505H**
$\Delta\Delta E_{DISP}$	-0.07	-0.68	-0.77	-0.49	-0.56	-1.01	-0.23	-0.42	-0.45	-0.37	-0.23	-0.34
$\Delta\Delta E_{ELE}$	-0.07	0.12	-1.46	-1.24	-0.68	-1.59	-0.52	-0.83	-1.31	-0.75	-0.21	0.12
$\Delta\Delta H$	-0.14	-0.56	-2.23	-1.73	-1.24	-2.60	-0.75	-1.25	-1.76	-1.12	-0.44	-0.22
$\Delta\Delta IE$	-0.05	-0.14	0.08	0.15	-0.10	0.19	0.03	-0.14	-0.17	-0.16	-0.04	0.01
$\boldsymbol{\Delta\Delta G_{CoV-2}}$	**-0.19**	**-0.70**	**-2.15**	**-1.58**	**-1.34**	**-2.41**	**-0.72**	**-1.39**	**-1.93**	**-1.28**	**-0.48**	**-0.21**
	**(0.16)**	**(0.09)**	**(0.10)**	**(0.11)**	**(0.16)**	**(0.07)**	**(0.12)**	**(0.09)**	**(0.13)**	**(0.16)**	**(0.16)**	**(0.08)**

	**Y505W**
$\Delta\Delta E_{DISP}$	0.15
$\Delta\Delta E_{ELE}$	-0.35
$\Delta\Delta H$	-0.20
$\Delta\Delta IE$	0.11
$\boldsymbol{\Delta\Delta G_{ACE2}}$	**-0.09**
	**(0.14)**

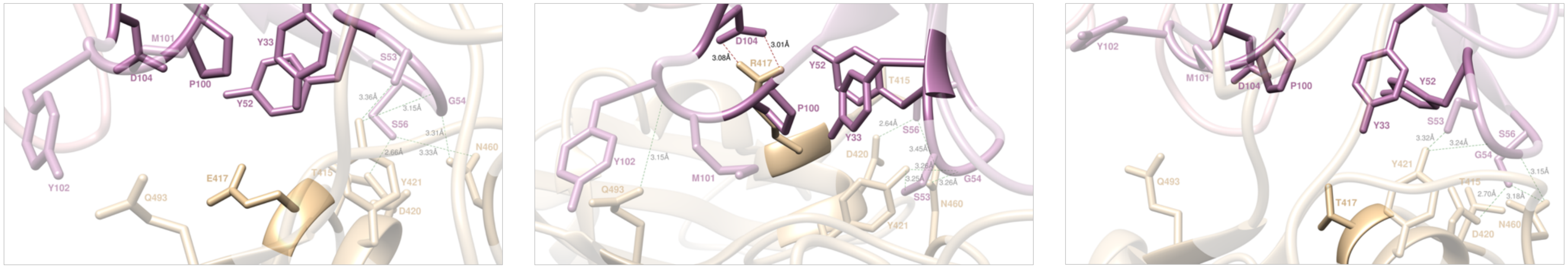

Fig. S11. Main interactions involving the S-RBD$_{CoV-2}$ E417 (left), R417 (middle), and T417 (right) at the interface with the LY-Cov016 antibody as obtained from the corresponding equilibrated MD simulations. The wild-type K417 and the N417 mutant complexes are presented and discussed in the main text (Figures 5D and 7A). In this and all remaining Figures, the secondary structure of the S-RBD$_{CoV-2}$ is shown as a light tan ribbon, while the heavy and light chains of the LY-Cov016 antibody are portrayed as light mulberry and light pink icing ribbons, respectively. Each protein residue under discussion and all other residues directly interacting with it are highlighted in dark matching-colored sticks and labelled; further residues/interactions related to the residue under investigation are evidenced in light matching-colored sticks and labelled in light gray. Hydrogen bonds and salt bridges directly involving the residue under discussion are represented as dark green and dark red broken lines, respectively, and the relevant average distances are reported (in black) accordingly. New HBs and SBs eventually detected in each mutant complex are also indicated using dark green/red broken lines and black labels. Further important HBs and SBs detected in each complex are also indicated using light green/red broken lines and light gray labels. For further details see Tables S15 and S19.

Table S19. Main intermolecular interactions between the wild-type S-RBD$_{CoV-2}$ residue K417 and all considered mutants* at the protein-protein interface detected during MD simulations of the RBD of SARS-CoV-2 (COV-2) in complex with the LY-Cov016 antibody. HB = hydrogen bond; SB = salt bridge; CI = contact interactions, including van der Waals/hydrophobic (vdW/h), polar (p), π/π and π/cation (π/c) interactions. In the HB column, s-s indicates side chain-side chain interactions while s-b or b-s indicate side chain-backbone or backbone-side chain interactions, respectively. Preserved/new or lost interactions are marked with the symbols ✓ and ✗, respectively. Relevant changes in the type/nature of the interactions are indicated in parenthesis. For HBs and SBs, the relevant average lengths (in Å) are also reported (their standard deviations, all within 10%, are not shown for clarity). *Mutant N417 is discussed in detail in main text.

**SB**	**COV-2**	**LY-Cov016**	**K417**	**E417**	**N417**	**R417**	**T417**
	X417	D_H104	✓(2.92,3.10)	✗	✗	✓(3.01,3.08)	✗
**HB**	**COV-2**	**LY-Cov016**	**K417**	**E417**	**N417**	**R417**	**T417**
s-s	T415	S_H56	✓(3.13)	✗	✗(p)	✗(p)	✗(p)
**HB**	**COV-2**	**LY-Cov016**	**K417**	**E417**	**N417**	**R417**	**T417**

s-s	X417	Y_H52	✓(2.98)	✗(vdW/h)	✗	✗(p)	✗
**HB**	**COV-2**	**LY-Cov016**	**K417**	**E417**	**N417**	**R417**	**T417**
s-s	D420	S_H56	✓(3.07)	✓(2.66)	✓(2.92)	✓(2.64)	✓(2.70)
**HB**	**COV-2**	**LY-Cov016**	**K417**	**E417**	**N417**	**R417**	**T417**
s-s	Y421	S_H53	✓(3.09)	✓(3.36)	✗(p)	✓(3.25)	✓(3.32)
s-b	Y421	G_H54	✓(3.21)	✓(3.15)	✓(3.20)	✓(3.26)	✓(3.24)
**HB**	**COV-2**	**LY-Cov016**	**K417**	**E417**	**N417**	**R417**	**T417**
s-b	N460	G_H54	✓(3.12)	✓(3.31)	✗	✓(3.26)	✓(3.15)
s-s	N460	S_H56	✓(3.04)	✓(3.33)	✓(3.41)	✓(3.45)	✓(3.18)
**HB**	**COV-2**	**LY-Cov016**	**K417**	**E417**	**N417**	**R417**	**T417**
s-b	Q493	Y_H102	✓(3.03)	✗(p)	✓(3.41)	✓(3.15)	✗
**CI**	**COV-2**	**LY-Cov016**	**K417**	E417	N417	R417	T417
p	X417	Y_H33	✓	✗	✗	✓	✗
vdW/h	X417	P_H100	✓	✗	✗	✓	✗
vdW/h	Y421	Y_H52	✓	✓	✓	✓	✓
p	Y421	Y_H33	✓	✓	✓	✓	✓
vdW/h	Q493	M_H101	✓	✓	✓	✓	✓
vdW/h	Q493	Y_H102	✓	✗	✓	✓	✓

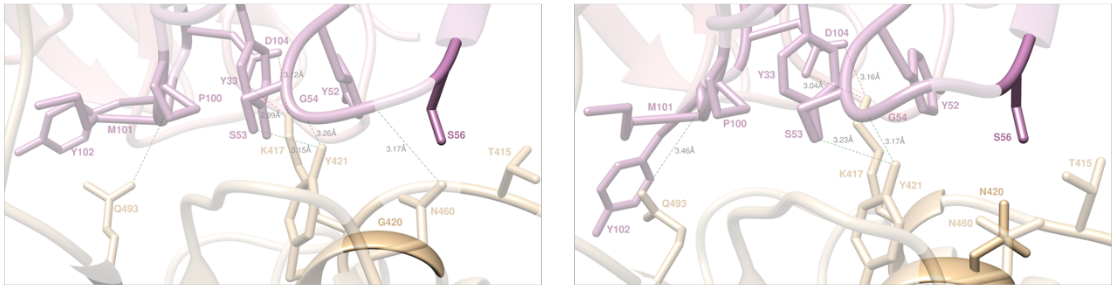

Fig. S12. Main interactions involving the S-RBD$_{CoV-2}$ G420 (left) and N420 (right) at the interface with the LY-Cov016 antibody as obtained from the corresponding equilibrated MD simulations. The wild type D420 and the A420 mutant complexes are presented and discussed in the main text (Figures 5D and 7B). Colors and other explanations as in Figure S11. For further details see Tables S15 and S20.

Table S20. Main intermolecular and intramolecular interactions between the wild-type S-RBD$_{CoV-2}$ residue D420 and all considered mutants* at the protein-protein interface detected during MD simulations of RBD of SARS-CoV-2 (COV-2) in complex with the LY-Cov016 antibody. Acronyms and other explanations as in Table S19. *Mutant A420 is discussed in detail in main text.

**SB**	**COV-2**	**LY-Cov016**	**D420**	**A420**	**G420**	**N420**
	K417	D_H104	✓(2.92,3.10)	✓(2.93,3.03)	✓(2.99,3.12)	✓(3.04,3.16)
**HB**	**COV-2**	**LY-Cov016**	**D420**	**A420**	**G420**	**N420**
s-s	T415	S_H56	✓(3.13)	✗	✗	✗(p)
**HB**	**COV-2**	**LY-Cov016**	**D420**	**A420**	**G420**	**N420**
s-s	K417	Y_H52	✓(2.98)	✗(p)	✗(p)	✗(p)
**HB**	**COV-2**	**LY-Cov016**	**D420**	**A420**	**G420**	**N420**
s-s	X420	S_H56	✓(3.07)	✗	✗	✗(p)
**HB**	**COV-2**	**LY-Cov016**	**D420**	**A420**	**G420**	**N420**
s-s	Y421	S_H53	✓(3.09)	✓(3.08)	✓(3.15)	✓(3.23)
s-b	Y421	G_H54	✓(3.21)	✓(3.25)	✓(3.26)	✓(3.17)
**HB**	**COV-2**	**LY-Cov016**	**D420**	**A420**	**G420**	**N420**

s-b	N460	G_H54	√(3.12)	✗	√(3.17)	✗
s-s	N460	S_H56	√(3.04)	√(3.40)	✗(p)	✗(p)
**HB**	**COV-2**	**LY-Cov016**	**D420**	**A420**	**G420**	**N420**
s-b	Q493	Y_H102	√(3.03)	√(3.11)	√(3.30)	√(3.46)
**CI**	**COV-2**	**LY-Cov016**	**D420**	**A420**	**G420**	**N420**
p	K417	Y_H33	√	√	√	√
vdW/h	K417	P_H100	√	√	√	√
vdW/h	K421	Y_H52	√	√	√	√
p	Y421	Y_H33	√	√	√	√
vdW/h	Q493	M_H101	√	√	√	√
vdW/h	Q493	Y_H102	√	√	√	√

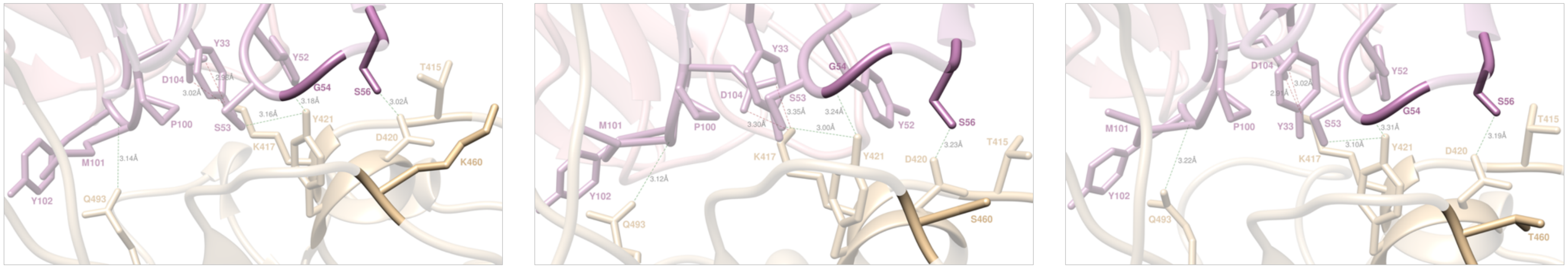

Fig. S13. Main interactions involving the S-RBD$_{CoV-2}$ K460 (left), S460 (middle), and T460 (right) at the interface with the LY-Cov016 antibody as obtained from the corresponding equilibrated MD simulations. The wild type N460 and the I460 mutant complexes are presented and discussed in the main text (Figures 5D and 7C). Colors and other explanations as in Figure S11. For further details see Tables S15 and S21.

Table S21. Main intermolecular and intramolecular interactions between the wild-type S-RBD$_{CoV-2}$ residue N460 and all considered mutants* at the protein-protein interface detected during MD simulations of RBD of SARS-CoV-2 (COV-2) in complex with the LY-Cov016 antibody. Acronyms and other explanations as in Table S19. *Mutant I460 is discussed in detail in main text.

**SB**	**COV-2**	**LY-Cov016**	**N460**	**I460**	**K460**	**S460**	**T460**
	K417	D$_H$104	✓(2.92,3.10)	✓(3.01,3.14)	✓(2.98,3.02)	✓(3.30,3.35)	✓(2.91,3.02)
**HB**	**COV-2**	**LY-Cov016**	**N460**	**I460**	**K460**	**S460**	**T460**
s-s	T415	S$_H$56	✓(3.13)	✗(p)	✗(p)	✗(p)	✗(p)
**HB**	**COV-2**	**LY-Cov016**	**N460**	**I460**	**K460**	**S460**	**T460**
s-s	K417	Y$_H$52	✓(2.98)	✗(p)	✗(p)	✗(p)	✗(p)
**HB**	**COV-2**	**LY-Cov016**	**N460**	**I460**	**K460**	**S460**	**T460**
s-s	D420	S$_H$56	✓(3.07)	✓(2.65)	✓(3.02)	✓(3.23)	✓(3.19)
**HB**	**COV-2**	**LY-Cov016**	**N460**	**I460**	**K460**	**S460**	**T460**
s-s	Y421	S$_H$53	✓(3.09)	✗(p)	✓(3.16)	✓(3.00)	✓(3.10)
s-b	Y421	G$_H$54	✓(3.21)	✓(3.17)	✓(3.18)	✓(3.24)	✓(3.31)
**HB**	**COV-2**	**LY-Cov016**	**N460**	**I460**	**K460**	**S460**	**T460**

s-b	X460	G_H54	✓(3.12)	✗	✗	✗	✗
s-s	X460	S_H56	✓(3.04)	✗	✗	✗	✗
**HB**	**COV-2**	**LY-Cov016**	**N460**	**I460**	**K460**	**S460**	**T460**
s-b	Q493	Y_H102	✓(3.03)	✓(3.10)	✓(3.14)	✓(3.12)	✓(3.22)
**CI**	**COV-2**	**LY-Cov016**	**N460**	**I460**	**K460**	**S460**	**T460**
p	K417	Y_H33	✓	✓	✓	✓	✓
vdW/h	K417	P_H100	✓	✓	✓	✓	✓
vdW/h	K421	Y_H52	✓	✓	✓	✓	✓
p	Y421	Y_H33	✓	✓	✓	✓	✓
vdW/h	Q493	M_H101	✓	✓	✓	✓	✓
vdW/h	Q493	Y_H102	✓	✓	✓	✓	✓

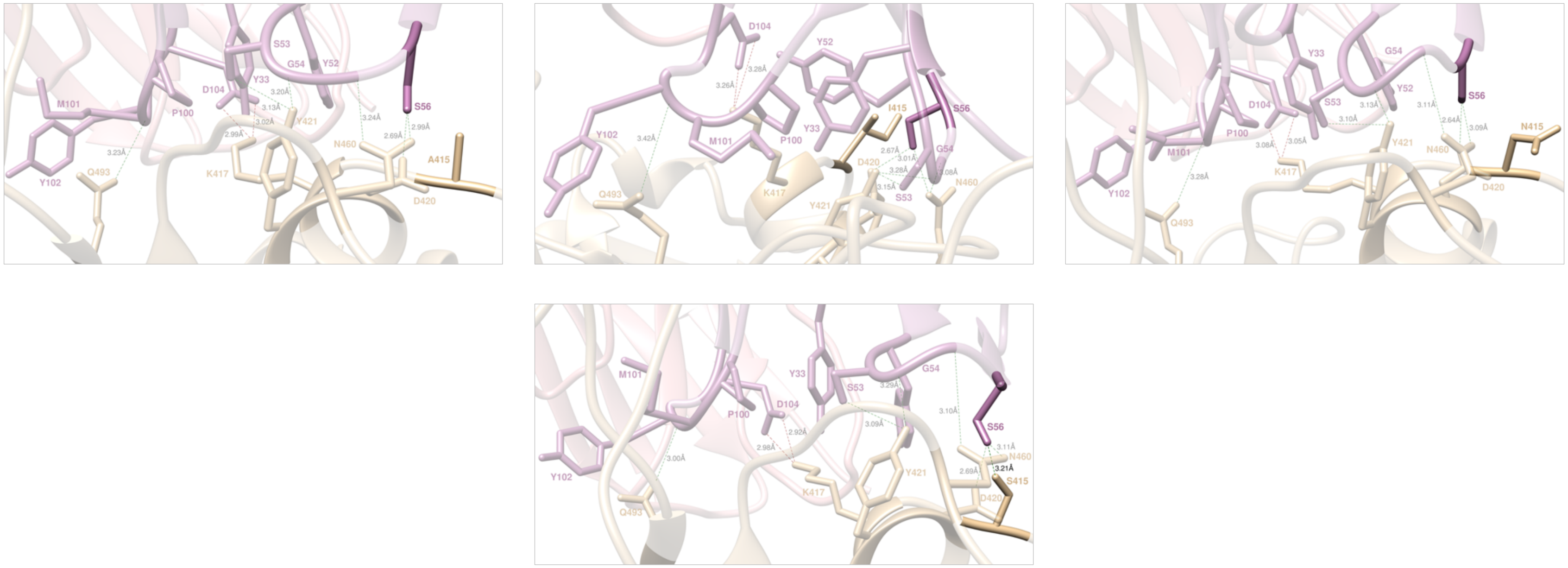

Fig. S14. Main interactions involving the S-RBD$_{CoV-2}$ A415 (top left), I415 (top middle), N415 (top right), and S415 (bottom) at the interface with LY-Cov016 as obtained from the corresponding equilibrated MD simulations. The wild type T415 and the P415 mutant complexes are presented and discussed in the main text (Figures 5D and 7D). Colors and other explanations as in Figure S11. For further details see Tables S15 and S22.

Table S22. Main intermolecular and intramolecular interactions between the wild-type S-RBD$_{CoV-2}$ residue T415 and all considered mutants* at the protein-protein interface detected during MD simulations of RBD of SARS-CoV-2 (COV-2) in complex with the LY-Cov016. Acronyms and other explanations as in Table S19. *Mutant P415 is discussed in detail in main text.

**SB**	**COV-2**	**LY-Cov016**	**T415**	**A415**	**I415**	**N415**	**P415**	**S415**
	K417	D_H104	✓(2.92,3.10)	✓(3.02,2.99)	✓(3.26,3.28)	✓(3.08,3.05)	✓(2.95,2.94)	✓(2.92,2.98)
**HB**	**COV-2**	**LY-Cov016**	**T415**	**A415**	**I415**	**N415**	**P415**	**S415**
s-s	X415	S_H56	✓(3.13)	✗	✗(vdW/h)	✗(p)	✗(vdW/h)	✓(3.21)
**HB**	**COV-2**	**LY-Cov016**	**T415**	**A415**	**I415**	**N415**	**P415**	**S415**

s-s	K417	Y_H52	✓(2.98)	✗(p)	✗(p)	✗(p)	✗(p)	✗(p)
**HB**	**COV-2**	**LY-Cov016**	**T415**	**A415**	**I415**	**N415**	**P415**	**S415**
s-s	D420	S_H56	✓(3.07)	✓(2.69)	✓(2.67)	✓(2.64)	✓(2.84)	✓(2.69)
**HB**	**COV-2**	**LY-Cov016**	**T415**	**A415**	**I415**	**N415**	**P415**	**S415**
s-s	Y421	S_H53	✓(3.09)	✓(3.13)	✓(3.15)	✓(3.10)	✓(3.16)	✓(3.09)
s-b	Y421	G_H54	✓(3.21)	✓(3.20)	✓(3.28)	✓(3.13)	✓(3.21)	✓(3.29)
**HB**	**COV-2**	**LY-Cov016**	**T415**	**A415**	**I415**	**N415**	**P415**	**S415**
s-b	N460	G_H54	✓(3.12)	✓(3.24)	✓(3.08)	✓(3.11)	✓(3.19)	✓(3.10)
s-s	N460	S_H56	✓(3.04)	✓(2.99)	✓(3.01)	✓(3.09)	✓(3.05)	✓(3.11)
**HB**	**COV-2**	**LY-Cov016**	**T415**	**A415**	**I415**	**N415**	**P415**	**S415**
s-b	Q493	Y_H102	✓(3.03)	✓(3.23)	✓(3.42)	✓(3.28)	✗(p)	✓(3.00)
**CI**	**COV-2**	**LY-Cov016**	**T415**	**A415**	**I415**	**N415**	**P415**	**S415**
p	K417	Y_H33	✓	✓	✓	✓	✓	✓
vdW/h	K417	P_H100	✓	✓	✓	✓	✓	✓
vdW/h	Y421	Y_H52	✓	✓	✓	✓	✓	✓
p	Y421	Y_H33	✓	✓	✓	✓	✓	✓
vdW/h	Q493	M_H101	✓	✓	✓	✓	✓	✓
vdW/h	Q493	Y_H102	✓	✓	✓	✓	✗	✓

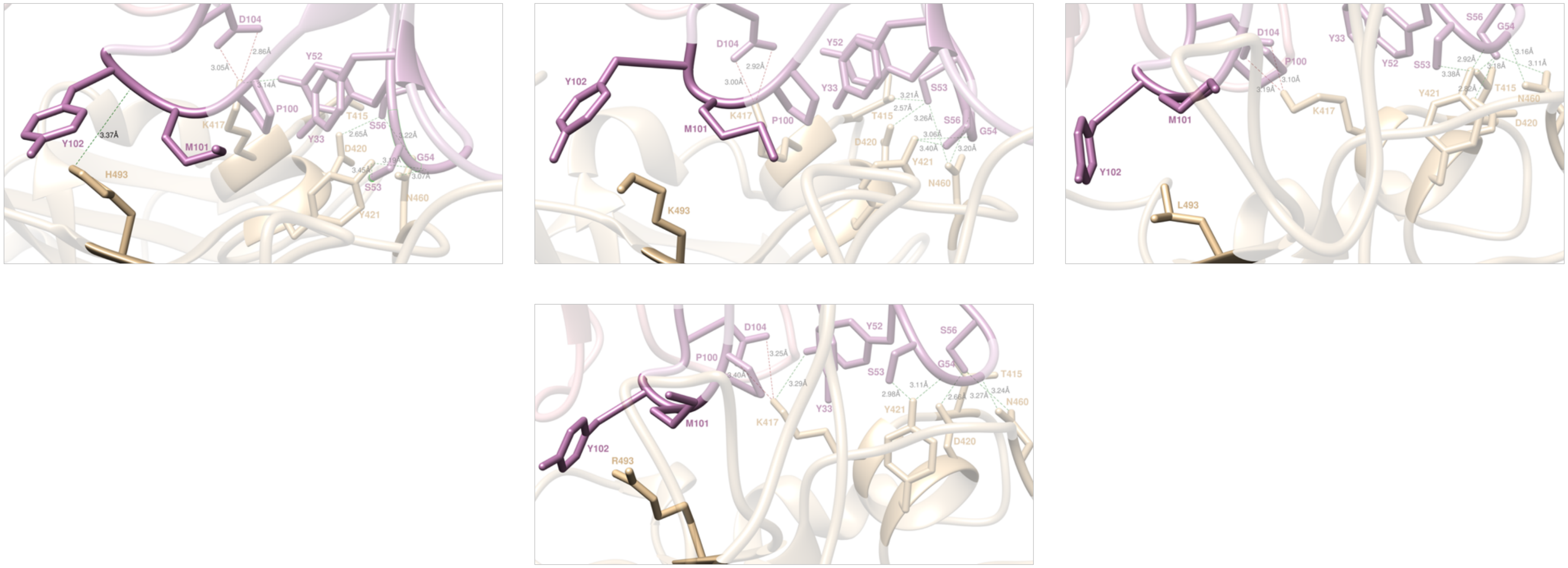

Fig. S15. Main interactions involving the S-RBD$_{CoV-2}$ H493 (top left), K493 (top middle), L493 (top right), and R493 (bottom) at the interface with LY-Cov016 as obtained from the corresponding equilibrated MD simulations. The wild-type Q493 is presented and discussed in the main text (Figure 5D). Colors and other explanations as in Figure S11. For further details see Tables S15 and S23.

Table S23. Main intermolecular interactions between the wild-type S-RBD$_{CoV-2}$ residue Q493 and all considered mutants at the protein-protein interface detected during MD simulations of the RBD of SARS-CoV-2 (COV-2) in complex with the LY-Cov016 antibody. Acronyms and other explanations as in Table S19.

**SB**	**COV-2**	**LY-Cov016**	**Q493**	**H493**	**K493**	**L493**	**R493**
	K417	D_H104	✓(2.92,3.10)	✓(3.05,2.86)	✓(2.92,3.00)	✓(3.19,3.10)	✓(3.40,3.25)
**HB**	**COV-2**	**LY-Cov016**	**Q493**	**H493**	**K493**	**L493**	**R493**
s-s	T415	S_H56	✓(3.13)	✗(p)	✓(3.21)	✓(3.18)	✗(p)
**HB**	**COV-2**	**LY-Cov016**	**Q493**	**H493**	**K493**	**L493**	**R493**

s-s	K417	Y_H52	√(2.98)	√(3.14)	✗(p)	✗(p)	√(3.29)
**HB**	**COV-2**	**LY-Cov016**	**Q493**	**H493**	**K493**	**L493**	**R493**
s-s	D420	S_H56	√(3.07)	√(2.65)	√(2.67)	√(2.82)	√(2.66)
**HB**	**COV-2**	**LY-Cov016**	**Q493**	**H493**	**K493**	**L493**	**R493**
s-s	Y421	S_H53	√(3.09)	√(3.45)	√(3.40)	√(3.38)	√(2.98)
s-b	Y421	G_H54	√(3.21)	√(3.19)	√(3.06)	√(2.92)	√(3.11)
**HB**	**COV-2**	**LY-Cov016**	**Q493**	**H493**	**K493**	**L493**	**R493**
s-b	N460	G_H54	√(3.12)	√(3.22)	√(3.20)	√(3.16)	√(3.24)
s-s	N460	S_H56	√(3.04)	√(3.07)	√(3.26)	√(3.11)	√(3.27)
**HB**	**COV-2**	**LY-Cov016**	**Q493**	**H493**	**K493**	**L493**	**R493**
s-b	X493	Y_H102	√(3.03)	√(3.37)	✗(p)	✗	✗(p)
**CI**	**COV-2**	**LY-Cov016**	**Q493**	**H493**	**K493**	**L493**	**R493**
p	K417	Y_H33	√	√	√	√	√
vdW/h	K417	P_H100	√	√	√	√	√
vdW/h	K421	Y_H52	√	√	√	√	√
p	Y421	Y_H33	√	√	√	√	√
vdW/h	X493	M_H101	√	√	√	√	√
vdW/h	X493	Y_H102	√	√	√	√	√

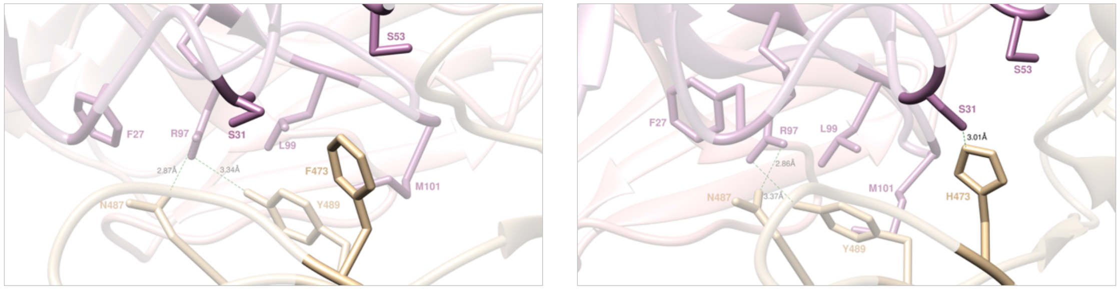

Fig. S16. Main interactions involving the S-RBD$_{CoV-2}$ F473 (left) and H473 (right) at the interface with the LY-Cov016 antibody as obtained from the corresponding equilibrated MD simulations. The wild-type Y473 is presented and discussed in the main text (Figure 5E). Colors and other explanations as in Figure S11. For further details see Tables S15 and S24.

Table S24. Main intermolecular interactions between the wild-type S-RBD$_{CoV-2}$ residue Y473 and all considered mutants at the protein-protein interface detected during MD simulations of the RBD of SARS-CoV-2 (COV-2) in complex with the LY-Cov016 antibody. Acronyms and other explanations as in Table S19.

**HB**	**COV-2**	**LY-Cov016**	**Y473**	**F473**	**H473**
s-s	X473	S_H31	✓(2.83)	✗(vdW/h)	✓(3.01)
**HB**	**COV-2**	**LY-Cov016**	**Y473**	**F473**	**H473**
s-s	N487	R_H97	✓(2.86)	✓(2.87)	✓(2.86)
**HB**	**COV-2**	**LY-Cov016**	**Y473**	**F473**	**H473**
s-s	Y489	R_H97	✓(3.38)	✓(3.34)	✓(3.37)
**CI**	**COV-2**	**LY-Cov016**	**Y473**	**F473**	**H473**
p	X473	S_H53	✓	✗(vdW/h)	✓
vdW/h	N487	F_H27	✓	✓	✓
vdW/h	Y489	L_H99	✓	✓	✓
vdW/h	Y489	M_H101	✓	✓	✓

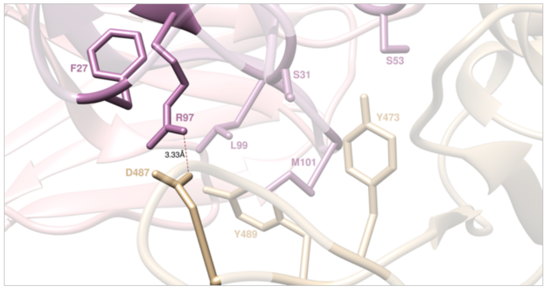

Fig. S17. Main interactions involving the S-RBD$_{CoV-2}$ D487 at the interface with the LY-Cov016 antibody as obtained from the corresponding equilibrated MD simulations. The wild-type N487 is presented and discussed in the main text (Figure 5E). Colors and other explanations as in Figure S11. For further details see Tables S15 and S25.

Table S25. Main intermolecular interactions between the wild-type S-RBD$_{CoV-2}$ residue N487 and all considered mutants at the protein-protein interface detected during MD simulations of the RBD of SARS-CoV-2 (COV-2) in complex with the LY-Cov016 antibody. Acronyms and other explanations as in Table S19.

**HB**	**COV-2**	**LY-Cov016**	**N487**	**D487**
s-s	Y473	S_H31	✓(2.83)	✗(p)
**HB**	**COV-2**	**LY-Cov016**	**N487**	**D487**
s-s	X487	R_H97	✓(2.86)	✓(SB,3.33)
**HB**	**COV-2**	**LY-Cov016**	N487	D487
s-s	Y489	R_H97	✓(3.38)	✗(p)
**CI**	**COV-2**	**LY-Cov016**	**N487**	**D487**
p	Y473	S_H53	✓	✓
vdW/h	X487	F_H27	✓	✗
vdW/h	Y489	L_H99	✓	✓
vdW/h	Y489	M_H101	✓	✓

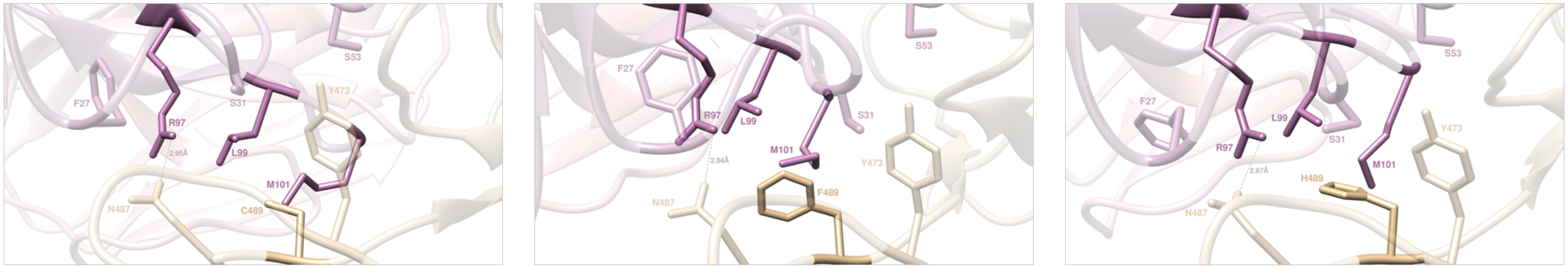

Fig. S18. Main interactions involving the S-RBD$_{CoV\text{-}2}$ C489 (top), F489 (middle), and H489 (right) at the interface with the LY-Cov016 antibody as obtained from the corresponding equilibrated MD simulations. The wild type Y489 and the S489 mutant complexes are presented and discussed in the main text (Figures 5E and 7E). Colors and other explanations as in Figure S11. For further details see Tables S15 and S26.

Table S26. Main intermolecular and intramolecular interactions between the wild-type S-RBD$_{CoV\text{-}2}$ residue Y489 and all considered mutants* at the protein-protein interface detected during MD simulations of RBD of SARS-CoV-2 (COV-2) in complex with the LY-Cov016 antibody. Acronyms and other explanations as in Table S19. *Mutant S489 is discussed in detail in main text.

HB	COV-2	LY-Cov016	Y489	C489	F489	H489	S489
s-s	Y473	$S_{H}31$	✓(2.83)	✗(p)	✗(p)	✗(p)	✗(p)
**HB**	**COV-2**	**LY-Cov016**	**Y489**	**C489**	**F489**	**H489**	**S489**
s-s	N487	$R_{H}97$	✓(2.86)	✓(2.95)	✓(2.84)	✓(2.87)	✓(2.99)
**HB**	**COV-2**	**LY-Cov016**	**Y489**	**C489**	**F489**	**H489**	**S489**
s-s	X489	$R_{H}97$	✓(3.38)	✗	✗(vdW/h)	✗(p)	✗
**CI**	**COV-2**	**LY-Cov016**	**Y489**	**C489**	**F489**	**H489**	**S489**
p	Y473	$S_{H}53$	✓	✓	✓	✓	✓
vdW/h	N487	$F_{H}27$	✓	✓	✓	✓	✓
vdW/h	X489	$L_{H}99$	✓	✗	✓	✓	✗
vdW/h	X489	$M_{H}101$	✓	✓	✓	✓	✓

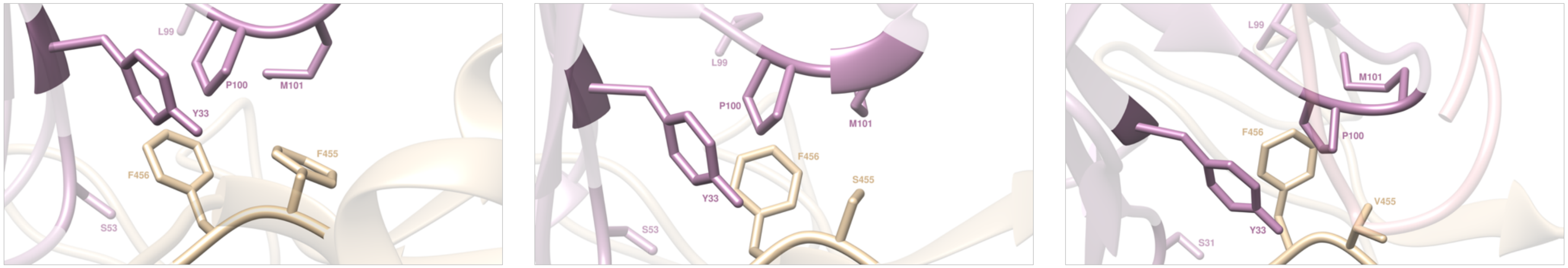

Fig. S19. Main interactions involving the S-RBD$_{CoV-2}$ F455 (left), S455 (middle), and V455 (right) at the interface with the LY-Cov016 antibody as obtained from the corresponding equilibrated MD simulations. The wild-type L455 is presented and discussed in the main text (Figure 5F). Colors and other explanations as in Figure S11. For further details see Tables S15 and S27.

Table S27. Main intermolecular interactions between the wild-type S-RBD$_{CoV-2}$ residue L455 and all considered mutants at the protein-protein interface detected during MD simulations of the RBD of SARS-CoV-2 (COV-2) in complex with the LY-Cov016 antibody. Acronyms and other explanations as in Table S19.

**CI**	**COV-2**	**LY-Cov016**	**L455**	**F455**	**S455**	**V455**
vdW/h	X455	Y_H33	✓	✓	✓	✓
vdW/h	X455	P_H100	✓	✓	✗	✓
vdW/h	X455	M_H101	✓	✓	✗	✗
**CI**	**COV-2**	**LY-Cov016**	**L455**	**F455**	**S455**	**V455**
vdW/h	F456	Y_H33	✓	✓	✓	✓
vdW/h	F456	S_H53	✓	✓	✓	✓
vdW/h	F456	L_H99	✓	✓	✓	✓
vdW/h	F456	P_H100	✓	✓	✓	✓
vdW/h	F456	M_H101	✓	✓	✓	✓

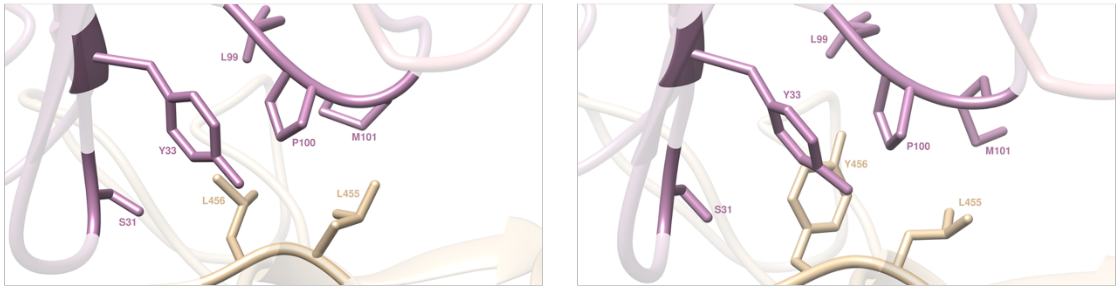

Fig. S20. Main interactions involving the S-RBD$_{CoV-2}$ L456 (left) and Y456 (right) at the interface with the LY-Cov016 antibody as obtained from the corresponding equilibrated MD simulations. The wild-type F456 is presented and discussed in the main text (Figure 5F). Colors and other explanations as in Figure S11. For further details see Tables S1 and S28.

Table S28. Main intermolecular interactions between the wild-type S-RBD$_{CoV-2}$ residue F456 and all considered mutants at the protein-protein interface detected during MD simulations of the RBD of SARS-CoV-2 (COV-2) in complex with the LY-Cov016 antibody. Acronyms and other explanations as in Table S19.

**CI**	**COV-2**	**LY-Cov016**	**F456**	**L456**	**Y456**
vdW/h	L455	Y_H33	✓	✓	✓
vdW/h	L455	P_H100	✓	✓	✓
vdW/h	L455	M_H101	✓	✓	✓
**CI**	**COV-2**	**LY-Cov016**	**F456**	**L456**	**Y456**
vdW/h	X456	Y_H33	✓	✓	✓
vdW/h	X456	S_H53	✓	✓	✓
vdW/h	X456	L_H99	✓	✗	✓
vdW/h	X456	P_H100	✓	✓	✓
vdW/h	X456	M_H101	✓	✓	✓

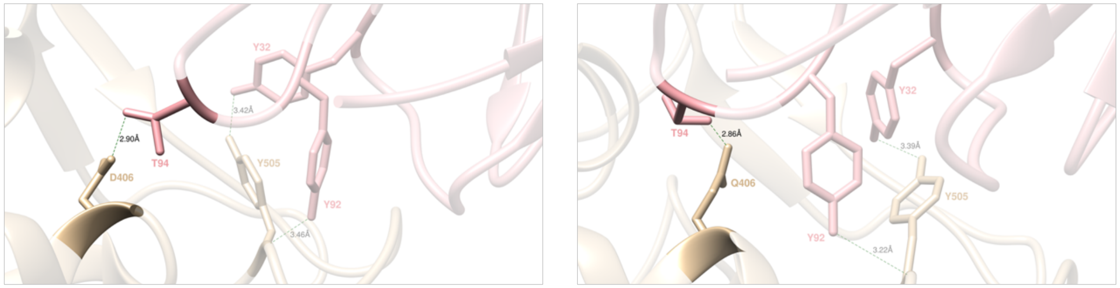

Fig. S21. Main interactions involving the S-RBD$_{CoV-2}$ D406 (left) and Q456 (right) at the interface with the LY-Cov016 antibody as obtained from the corresponding equilibrated MD simulations. The wild-type E406 is presented and discussed in the main text (Figure 5G). Colors and other explanations as in Figure S11. For further details see Tables S15 and S29.

Table S29. Main intermolecular interactions between the wild-type S-RBD$_{CoV-2}$ residue D406 and all considered mutants at the protein-protein interface detected during MD simulations of the RBD of SARS-CoV-2 (COV-2) in complex with the LY-Cov016 antibody. Acronyms and other explanations as in Table S19.

**HB**	**COV-2**	**LY-Cov016**	**E406**	**D406**	**Q406**
s-s	X406	T_L94	✓(2.84)	✓(2.90)	✓(2.86)
**HB**	**COV-2**	**LY-Cov016**	**E406**	**D406**	**Q406**
s-s	Y505	Y_L32	✓(3.01)	✓(3.42)	✓(3.39)
b-s	Y505	Y_L92	✓(3.14)	✓ (3.46)	✓(3.22)

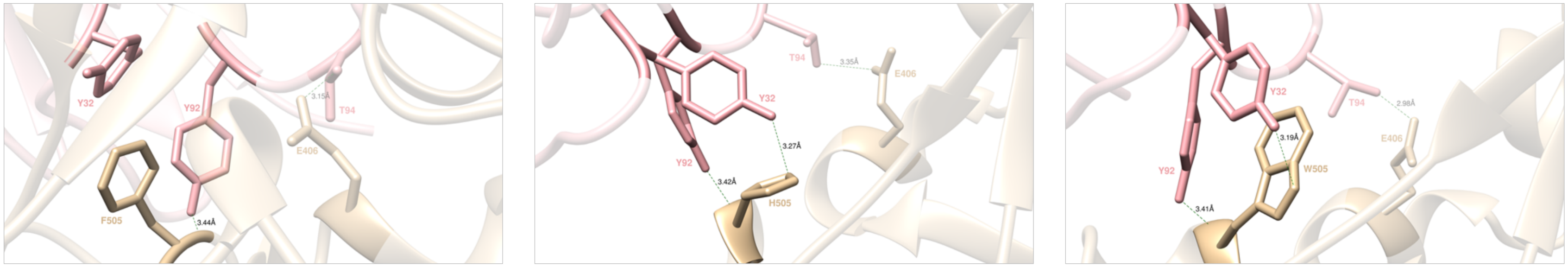

Fig. S22. Main interactions involving the S-RBD$_{CoV-2}$ F505 (left), H505 (middle), and W505 (right) at the interface with the LY-Cov016 antibody as obtained from the corresponding equilibrated MD simulations. The wild-type Y505 is presented and discussed in the main text (Figure 5G). Colors and other explanations as in Figure S11. For further details see Tables S15 and S30.

Table S30. Main intermolecular interactions between the wild-type S-RBD$_{CoV-2}$ residue Y505 and all considered mutants at the protein-protein interface detected during MD simulations of the RBD of SARS-CoV-2 (COV-2) in complex with the LY-Cov016 antibody. Acronyms and other explanations as in Table S19.

**HB**	**COV-2**	**LY-Cov016**	**Y505**	**F505**	**H505**	**W505**
s-s	E406	T_L94	✓(2.84)	✓(3.15)	✓(3.35)	✓(2.98)
**HB**	**COV-2**	**LY-Cov016**	**Y505**	**F505**	**H505**	**W505**
s-s	X505	Y_L32	✓(3.01)	✗(π/π)	✓(3.27)	✓(3.19)
b-s	X505	Y_L92	✓(3.14)	✓(3.44)	✓(3.42)	✓(3.41)
